# Climate Adaptation and Drift Shape the Genomes of Two Eel-Goby Sister Species Endemic to Contrasting Latitude

**DOI:** 10.3390/ani13203240

**Published:** 2023-10-17

**Authors:** Zhenming Lü, Tianwei Liu, Yantao Liu, Yuzhen Wang, Jing Liu, Bingjian Liu, Li Gong, Liqin Liu

**Affiliations:** 1 National Engineering Laboratory of Marine Germplasm Resources Exploration and Utilization, College of Marine Sciences and Technology, Zhejiang Ocean University, Zhoushan 316022, China; nblzmnb@zjou.edu.cn (Z.L.); liutianwei@zjou.edu.cn (T.L.); liuyantao@zjou.edu.cn (Y.L.); 2022157@zjou.edu.cn (J.L.); liubingjian@zjou.edu.cn (B.L.); gongli@zjou.edu.cn (L.G.); 2 National Engineering Research Center for Facilitated Marine Aquaculture, Zhejiang Ocean University, Zhoushan 316022, China; wangyuzhen@zjou.edu.cn

**Keywords:** *O. lacepedii*, *O. rebecca*, thermal adaptation, ecological divergence, demographic processes

## Abstract

**Simple Summary:**

Climate is one of the most important drivers for adaptive evolution and ecological speciation in teleost species. Using whole-genome re-sequencing technology, this study evaluated the role of divergent climate selection in shaping the genetic divergence of two eel-goby sister species spanning contrasting latitude gradients. High baseline genetic differentiation was found between them. Substantial elevated divergent loci were detected to be widespread throughout their genomes, which may arise from divergent climate selection, in addition to historical demographic processes. The loci exhibiting selective signatures highlighted the roles of genes associated with substance metabolism, energy production, response to environmental cues, and reproductive activity in latitude gradient adaptation and hence the climate-driven divergence of the two species. The results provided important insights into how climate changes related to latitude may drive genetic differentiation and thereby ecological speciation in marine teleosts.

**Abstract:**

Deciphering the role of climate adaptation in generating genetic divergence and hence speciation is a central question in evolution. Comparisons of genomes of closely related species spanning selective climate gradients are particularly informative in discerning the signatures of selection and thereby providing valuable information concerning the role of climate adaptation in speciation. Here we re-sequenced 99 genomes of the two sister eel-goby species *Odontamblyopus lacepedii* and *O. rebecca*, which are endemic to tidal mudflats spanning contrasting latitude gradients, to estimate the influence of divergent climate selection on shaping genome-wide patterns of divergence. The results indicated that genome-wide differentiation between the two species was evident (genome-wide *FST* = 0.313). Against a background of high baseline genomic divergence, 588 and 1202 elevated divergent loci were detected to be widespread throughout their genomes, as opposed to focused within small islands of genomic regions. These patterns of divergence may arise from divergent climate selection in addition to genetic drift acting through past glacial segregation (1.46 million years ago). We identified several candidate genes that exhibited elevated divergence between the two species, including genes associated with substance metabolism, energy production, and response to environmental cues, all putative candidates closely linked to thermal adaptation expected from the latitude gradient. Interestingly, several candidates related to gamete recognition and time of puberty, and also exhibited elevated divergence, indicating their possible role in pre-zygote isolation and speciation of the two species. Our results would expand our knowledge on the roles of latitude climate adaptation and genetic drift in generating and maintaining biodiversity in marine teleosts.

## 1. Introduction

Climate adaptation has long been recognized as an important driver of speciation for its role in the development of intrinsic barriers to gene flow [1] in a process termed ecological speciation [2]. Under a scenario of climate-driven ecological speciation, adaptation to a distinct climate will usually lead to differentiation in small portions of loci (also referred to as “genomic islands”) responsible for adaptation or even fixation of different alleles in isolated populations at the incipient stage of speciation [3]. Afterward, such differentiation of the genes underlying these adaptations may gradually extend to the wider genome through linkage disequilibrium, to form reproductive incompatibility between species [4]. In such a scenario of ecological speciation, both adaptive divergence and reproductive isolation are products of divergent climate adaptations, even in the presence of recurrent gene flow [5]. However, both the prevalence and the genetic mechanisms underlying this pattern of speciation remain largely unknown.

Fortunately, dramatic progress in genome sequencing technology in the past decade has made these questions answerable using whole-genome sequencing in natural populations. The genomics era holds promise for research of ecological speciation, as it enables the detection of ecologically driven signatures of selection on a genome-wide scale [6]. One frequently used approach is to compare genomic patterns of differentiation between two ecologically divergent taxa spanning a selective climate gradient, a process usually referred to as genome scan, to identify genomic regions that would not conform to expectations based on neutral demographic models [6]. These regions are thereby recognized candidates under selection, and can be explored in further detail to identify the precise target and mode of the adaptive selection involved in species divergence and reproductive isolation. Closely diverged taxa are especially preferred in such genome scan systems, because early-acting barriers to gene flow usually have larger effects on driving reproductive incompatibility than late-acting barriers [7]. In addition, identifying the potential genomic regions under selection is easier when baseline differentiation is low, as expected for phylogenetically closely related taxa [8,9]. In recent years, such genomic-comparing methodology has been increasingly applied to non-model systems and has largely expanded our knowledge of the genetic mechanisms underlying the climate-driven adaptation and speciation in a range of taxa [10,11,12]. However, it is still less clear whether such climate-driven adaptation is also important for genetic differentiation and speciation within marine taxa, such as in teleost fishes.

Here we re-sequenced 99 genomes and investigated patterns of genome-wide divergence between two closely related mudflat endemics, *O. lacepedii* and *O. rebecca* [13,14], spanning large contrasting latitude gradients of China to estimate the influence of selective processes of the climate in shaping genome-wide patterns of divergence. *O. lacepedii* and *O. rebecca* are two sub-economically important sister species belonging to the genus *Odontamblyopus*, Family Gobiidae of Gobiiformes [13]. Although long considered as a single species, *O. lacepedii* and *O. rebecca* are currently recognized as two sister species that are parapatrically distributed along the coast of China, with *O. lacepedii* endemic to the north and *O. rebecca* in the south, leaving a narrow contact zone locating around the Taiwan strait regions [13]. The prevailing evolutionary hypothesis [13] suggests that these two sister species originated from a common ancestor with populations spanning the full range of the China coast and were split by the Pleistocene glaciation, resulting in population differentiation and speciation. After the recession of the Pleistocene ice mass, they secondarily contacted, and established the current ranges and ecotypes within this species complex [13]. While both species inhabit tidal mudflat habitats, variation in latitude affinity may suggest the role of divergent selection and climate adaptation in the speciation of these two *Odontamblyopus* gobies. Alternatively, changes in effective population size during ice-age isolation and postglacial re-colonization may have built up the role of genetic drift in driving interspecific differentiation. However, to date, the genome-wide pattern of differentiation and the divergent signature of climate selection between *O. lacepedii* and *O. rebecca*, have never been investigated. An exploration of the genomic landscape and genome-wide adaptive selection in these taxa will increase our understanding of how climate adaptations mediated through latitude gradients could drive the genome-wide divergence and hence the ecological speciation in marine teleost.

## 2. Materials and Methods

### 2.1. Sample Collection and DNA Isolation

**Figure 1:**
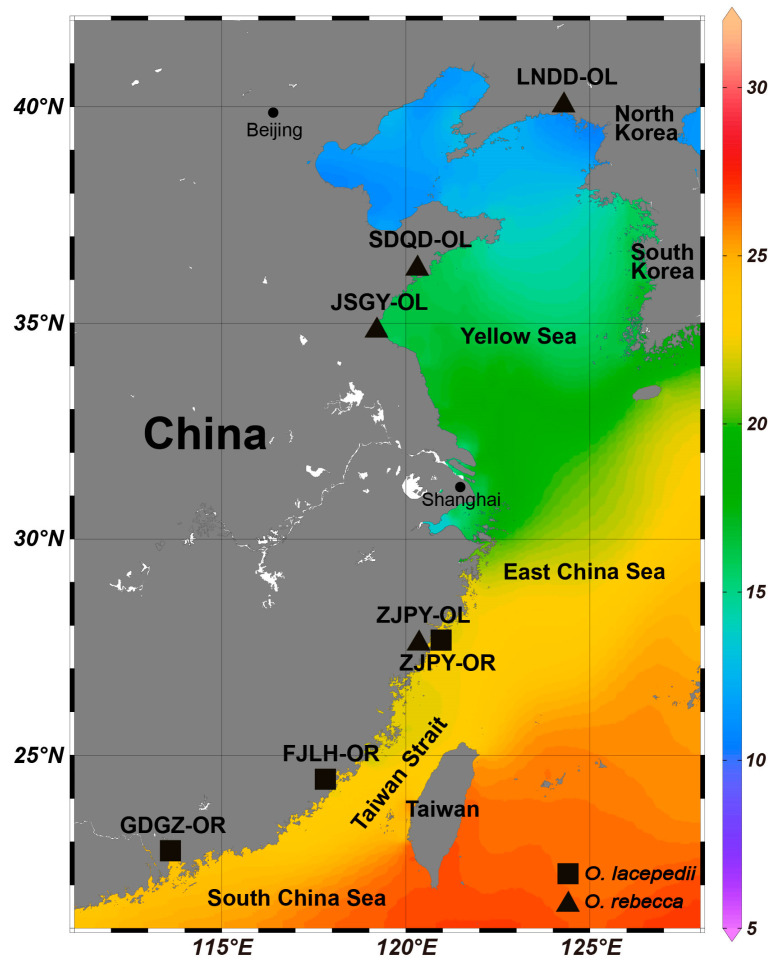
Sampling localities of two sister species of eel-goby, *Odontamblyopus lacepedii* and *O. rebecca* along the China coast in this study: ▲ and ■ represent the localities where *O. lacepedii* and *O. rebecca* were sampled, respectively. LNDD, SDQD, JSGY, ZJPY, FJLH, GDGZ represent samples collected from Dandong (Liaoning province), Qingdao (Shandong province), Ganyu (Jiangsu province), Pingyang (Zhejiang province), Longhai (Fujian province) and Guangzhou (Guangdong province), respectively. Different colors in the figure represent the yearly averaged surface water temperature in different sampling localities.

We collected fish samples through bottom trawling by local fishing boats, from four and three localities spanning the natural distributional ranges of *O. lacepedii* and *O. rebecca*, respectively (Figure 1; Table 1), including two parapatric populations in the contact zone near the Pingyang ocean regions. A total of 61 individuals of *O. lacepedii* and 38 individuals of *O. rebecca* were collected and the muscle tissues were immediately sampled after their death and stored in liquid nitrogen prior to DNA extraction. Genomic DNA was isolated from muscle tissues using a Blood and Cell Culture DNA Mini Kit (QIAGEN, Cat. No. 13343). The quality and concentration the of extracted DNA were assessed using a Pultton DNA/Protein Analyzer (Plextech, New York, NY, USA) and stored at −80 °C until use. All tissue collection and DNA isolation procedures conform to all relevant ethical regulations provided by the Institutional Animals Care and Use Committee of Zhejiang Ocean University.

### 2.2. Whole-Genome Resequencing and SNP Calling

A 150 bp paired-end sequencing library with a 350 bp insert size was constructed for every *O. lacepedii* and *O. rebecca* specimen using the TruSeq Library Construction Kit (Illumina, San Diego, CA, USA) and sequencing was performed from high-quality DNA based on the standard DNBSEQ-T7 platform (MGI Technology Co., Ltd., Shenzhen, China) with an expected target coverage of 25×. The resulting raw sequence data were deposited at the NCBI in the sequence read archive (SRA) under accession number (BioProject Number: PRJNA1006622). After quality control of the sequencing data using Trimmomatic (version 0.32) [15] to remove adapter sequences and low-quality bases from the start or the end of reads (base quality ≤ 20), the clean reads of each library were separately mapped to our previously sequenced reference genome of *O. rebecca* (GenBank: GCA_030686955.1) using BWA (version 0.7.12) with the parameter of: mem -t 4 -k 32 -M -R) [16]. In addition, SAMtools (version 0.1.19) [17] was applied to calculate the sequencing coverage and depth, and the potential PCR duplications were removed using the SAMtools command “rmdup”. SNPs calling was performed using the Haplotype Caller protocol in Genome Analysis Toolkit (GATK, version 4.1.9.0) software [18]. In order to guarantee the reliability of the SNPs data called, we removed those SNPs with low frequency of allele (MAF < 0.05) and low coverage (<10×) at the population level using GATK Variant Filtration. The remaining SNP after filtration were thereafter used for further analysis.

### 2.3. Population Genomics and Demographic Inference

We calculated genome-wide genetic diversity statistics (e.g., percentage of polymorphic loci (%), heterozygosity observed (Ho), heterozygosity expected (He), and nucleotide diversity (Pi)) within both species of *O. lacepedii* and *O. rebecca.* Clustering analyses were performed to visualize the population structure of *O. lacepedii* and *O. rebecca* using ADMIXTURE (version 1.3) [19]. To this end, sites with less than 10% of their data missing were used, and the number of coancestry clusters (K) ranged from 1 to 6. In addition, principal component analysis (PCA) was performed using the smartPCA program in the Eigensoft v5.0 package (Eigensoft, RRID:SCR 004965) [20] with all SNPs. In order to also adopt a phylogenetic perspective, neighbor-joining (NJ) trees were further constructed from the whole genome-wide SNPs using TreeBest software (version 1.9.2) (http://treesoft.sourceforge.net/treebest.shtml (accessed on 13 July 2023)), employing a rapid bootstrap procedure of 1000 replicates. To infer the demographic history of *O. lacepedii* and *O. rebecca*, genome-wide interspecific differentiation was evaluated with the FST statistics, calculated in non-overlapping 1 kb windows using the fst-sliding.pl script integrated in PoPoolation (version 1.2.2) [21]. The historical change of effective population size was analyzed employing pairwise sequentially Markovian coalescent (PSMC, https://github.com/lh3/psmc (accessed on 19 July 2023)) with the parameters of default. Here, we set the generation time (g) as 1.0 years, and the nucleotide mutation rate (μ) of the two species was 0.4 × 10^−8^ mutations per site per generation [22]. The divergence time and gene flow between the two species were further estimated using dadi software (version 1.6.3) [23], which takes past population size changes into account, via seven models (Model 1. divergence with no migrations (NM); Model 2. divergence with continuous symmetric migrations (SM); Model 3. divergence with continuous asymmetric migrations (AM); Model 4. divergence with symmetric migrations following secondary contact (SMSC); Model 5. divergence with asymmetric migrations following secondary contact (AMSC); Model 6. divergence with ancient symmetrical migration (ASM); Model 7. divergence with ancient asymmetrical migration (AAM)) to evaluate which model was the best (the model with lowest log-likelihood and AIC value), and was thereafter further used to calculate our results.

### 2.4. Detection of Selective Signatures

To fully evaluate genome-wide patterns of divergence and to identify the divergent signature of climate adaptation between *O. lacepedii* and *O. rebecca*, a combined approach was employed to define candidate-selected regions by involving both the Fst and genetic diversity (Pi) indexes. Firstly, we calculated the Fst value by sliding windows of 100 kb (a step of 10 kb) across the genome using VCFtools (version 0.1.15) [24] to identify candidate-selected regions if they contained window-based Fst estimates above the 95th percentile of the empirical distribution. Secondly, the Pi value in the same 100 kb windows was estimated to define windows above the 95th percentile of the Pi distribution. Overlapped windows with the top 5% of Fst and log2 (Pi) values were thereby recognized as the candidate-selected regions. The candidate-selected regions were then mapped to corresponding SNPs and genes. The positively selected genes (PSGs) were also used for GO and KEGG enrichment analysis via Enrich GO and Rscript. Significantly over-represented GO terms and KEGG pathways were identified with *p*-values < 0.05.

## 3. Results

To analyze the genetic diversity and divergence among the two sister species of *O. lacepedii* and *O. rebecca*, we conducted genome re-sequencing for a sample of 99 individuals collected from seven localities spanning a large latitude gradient of China coast (Figure 1; Table 1). In total, 16,225,922,040 clean reads were obtained and then mapped to the reference genome of *O. rebecca.* The results revealed highly conserved genomes of the two *Odontamblyopus* gobies, and there were 99.37% and 99.01% (Table S1) of the obtained sequences in *O. lacepedii* and *O. rebecca* that could be mapped to the reference genome, respectively. The mean coverage of each site reached a mean depth of 26.83× in the mapped regions of all the *O. lacepedii* and *O. rebecca* samples (Table S1). After a filtration and strict quality control process, a total of 9,972,482 high-quality SNP sites were generated across all the *O. lacepedii* and *O. rebecca* samples, and they are used for the downstream analyses (Table S1).

### 3.1. Genome-Wide Diversity and Genetic Divergence between the Two Sister Species

**Figure 2:**
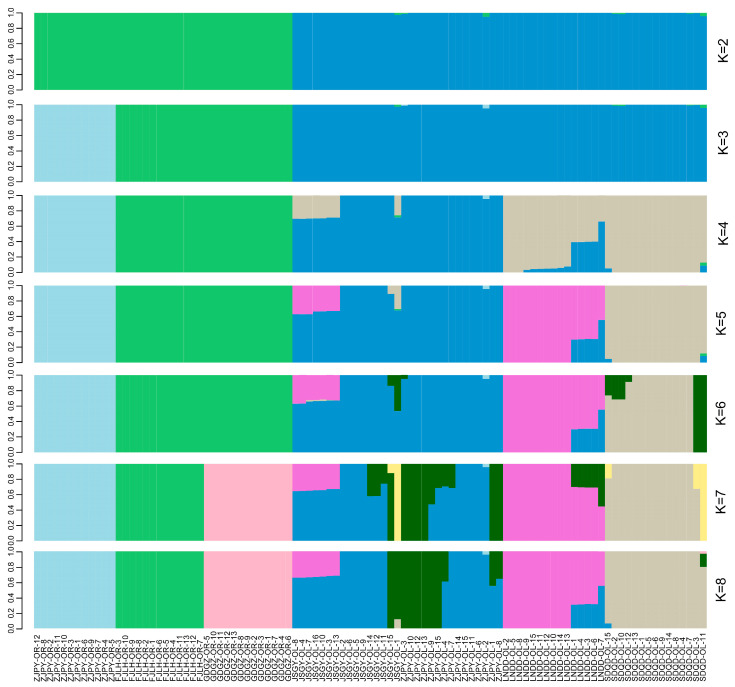
Genetic structure of *Odontamblyopus lacepedii* and *O. rebecca* inferred using ADMIXTURE. Each bar represents an individual, with different colors corresponding to one of the K ancestry clusters and length proportional to the assignment to that particular cluster. Individuals are grouped according to the location of sampling (LNDD, SDQD, JSGY, ZJPY, FJLH, GDGZ represent samples collected from Dandong (Liaoning province), Qingdao (Shandong province), Ganyu (Jiangsu province), Pingyang (Zhejiang province), Longhai (Fujian province) and Guangzhou (Guangdong province), respectively), with OL and OR indicating *O. lacepedii* and *O. rebecca* populations, respectively.

**Figure 3:**
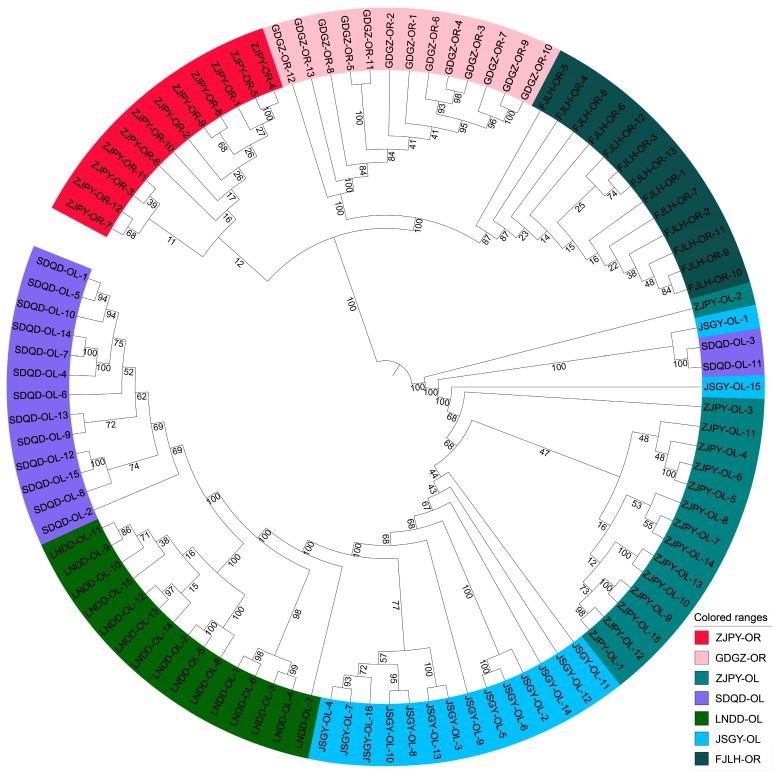
Neighbor-joining tree constructed from the allele-shared matrix of SNPs among *O. lacepedii* and *O. rebecca*. Sampling sites are colored according to the population to which they belong. Individuals are labeled according to the location of sampling as coded in Table 1 (LNDD, SDQD, JSGY, ZJPY, FJLH, GDGZ represent samples collected from Dandong (Liaoning province), Qingdao (Shandong province), Ganyu (Jiangsu province), Pingyang (Zhejiang province), Longhai (Fujian province) and Guangzhou (Guangdong province), respectively), with OL and OR indicating *O. lacepedii* and *O. rebecca* populations.

**Figure 4:**
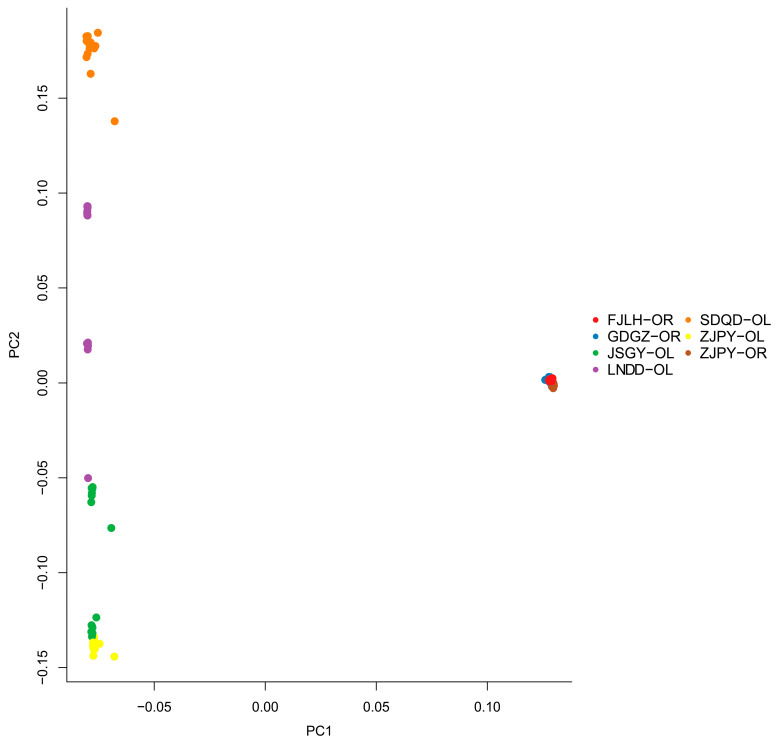
Principal component analysis (PCA) showing genetic distance among samples of *O. lacepedii* and *O. rebecca*. Sampling sites are colored according to the population to which they belong. Individuals are labeled according to the location of sampling as coded in Table 1 (LNDD, SDQD, JSGY, ZJPY, FJLH, GDGZ represent samples collected from Dandong (Liaoning province), Qingdao (Shandong province), Ganyu (Jiangsu province), Pingyang (Zhejiang province), Longhai (Fujian province) and Guangzhou (Guangdong province), respectively), with OL and OR indicating *O. lacepedii* and *O. rebecca* populations.

Genome-wide estimates of diversity varied slightly between the two sister species involved in this study (Table 1). In general, *O. rebecca* showed higher diversity in parameters, such as percentage of polymorphic loci (%), nucleotide diversity (Pi), heterozygosity observed (Ho), and heterozygosity expected (He) than *O. lacepedii*, consistent with the current “southern richness” theory [25]. Estimates of divergence showed a high averaged genome-wide Fst (0.313) across all autosomal SNPs, suggesting high baseline differentiation between them. Such high genome-wide differentiation was also confirmed by the afterward admixture analyses, which subdivided all genotyped samples into two species-specific clades when the number of clusters (K) was 2 in optimal (Figure 2; Figure S1). Further population sub-structuring was observed in the species of *O. lacepedii* when K = 3, in which individuals of the Pingyang (ZJPY) population were inferred to subdivide from the other populations, indicating slight clinal variation with latitude. However, no further reliable population sub-division was observed when K is above 4 (Figure 2). A neighbor-joining (NJ) tree further lent support for these differentiation patterns, with different geographical locations from the *O. lacepedii* and *O. rebecca* reflecting the grouping of populations (Figure 3). A principal component analysis (PCA) further supported these results, with the first two components explaining 69.96% and 2.14% of total genetic variance according to a Tracy–Widom test, respectively (Figure 4). Among the total number of polymorphisms, we identified 900,408 fixed SNPs between the two species, which accounted for 9.03% of the total polymorphisms and appeared, in general, to be uniformly distributed across the full genomic regions.

### 3.2. Demographic History of the Two Sister Species

**Figure 5:**
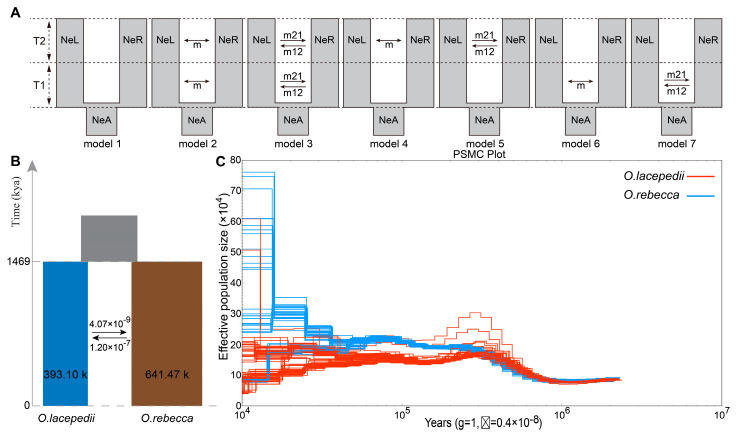
Coalescence analyses of demography in *O. lacepedii* and *O. rebecca* based on dadi software. (**A**) Seven models were tested, either assuming divergence with no migrations, or with continuous symmetric or asymmetric migrations migration across the genome. Model 1. divergence with no migrations; Model 2. divergence with continuous symmetric migrations; Model 3. divergence with continuous symmetric migrations; Model 4. divergence with symmetric migrations following secondary contact; Model 5. divergence with asymmetric migrations following secondary contact; Model 6. divergence with ancient continuous symmetrical migration; Model 7. divergence with ancient continuous asymmetrical migration; NeL, NeR, and NeA represent the effective population size of *O. lacepedii*, *O. rebecca* and their ancestor; m represent the migration direction. (**B**) The demographic process inferred from divergence with symmetric migrations following the secondary contact model, which is best fit for *O. lacepedii* and *O. rebecca* based on dadi analysis. Joint past population is in gray, *O. lacepedii* in blue and *O. rebecca* in brown. Estimated effective population sizes and the migration rate in genetic replacements per generation are indicated. (**C**) The population history estimated for *O. lacepedii* and *O. rebecca* based on PSMC software. The red and blue lines represent the population size changes in *O. lacepedii* and *O. rebecca*, respectively.

Reconstruction of demographic history is crucial for understanding the likely driving factors underlying the population differentiation and hence speciation in the two *Odontamblyopus* gobies. To infer the divergence time between *O. lacepedii* and *O. rebecca*, we used a method based on a diffusion approximation to the site frequency spectrum which takes past population size changes into account, and the result favored a demographic history of divergence with asymmetrical gene flow following divergence (Table 2), with a substantially higher migration rate in *O. rebecca*- to—*O.lacepedii* direction than the reverse (Figure 5). Based on this demographic scenario, the ancestors of the two gobies diverged into *O. lacepedii* and *O. rebecca* populations approximately 146.88 million years ago (Mya) (Figure 5B), which falls into a major glaciation period of Donau (~1.3–1.5 Ma) in the middle Pleistocene. The current effective population sizes (Ne) of *O.lacepedii* and *O.rebecca* are 393,102 and 641,470, respectively (Figure 5B). Both of the two species have experienced considerably long periods of population size expansion following divergence. However, they differed slightly in the expansion profile with *O. rebecca* experiencing population expansion after divergence and continuing up to the present (Figure 5C), whereas *O. lacepedii* experienced a population expansion followed by a substantially long period of a bottleneck after the last glacier (around 0.07 Mya) (Figure 5C). We found evidence of little continuous gene flow between *O. rebecca* and *O. lacepedii* after their divergence (Figure 5B). The migration rate (m) was also quite restricted between the two species, with the generation migration rate from *O. rebecca* into *O. lacepedii* being 1.20 × 10^−7^ genetic replacement per generation, and 4.07 × 10^−9^ genetic replacement from the reverse (Figure 5B), indicating an asymmetric quite low gene flow after the divergence between the two species.

### 3.3. Selective Signature in Genome of the Two Sister Species

**Figure 6:**
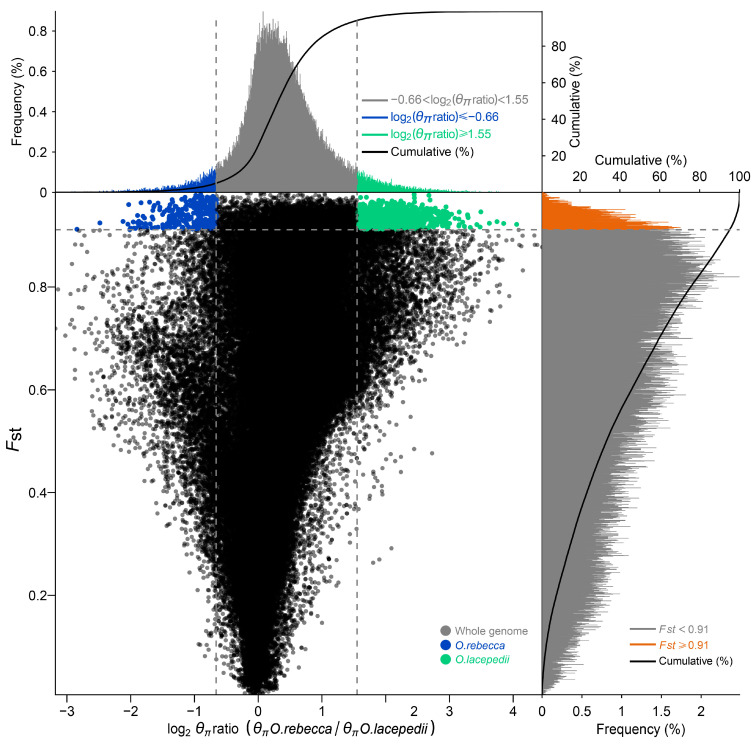
Distribution of log2 (Pi) ratios (*O. lacepedii*/*O. rebecca*) and Fst values, which are calculated in 100 kb windows sliding in 10 kb steps. Data points located to the left and right of the vertical dashed lines (corresponding to the 5% left and right tails of the empirical log2 (Pi) ratio distribution), and above the horizontal dashed line (the 5% right tail of the empirical FST distribution) were identified as selected regions for the *O. lacepedii* (green points) and *O. rebecca* (blue points) populations, respectively.

**Figure 7:**
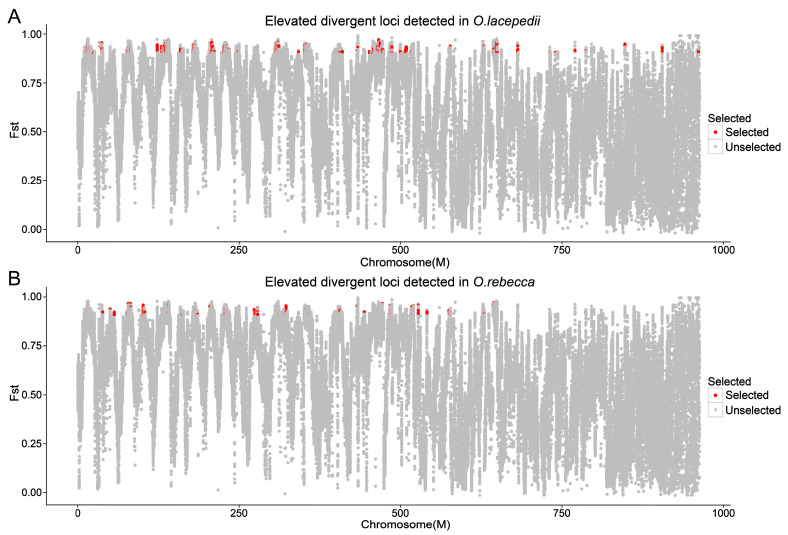
Genome-wide patterns of divergence and elevated divergent loci detected in *O. lacepedii* and *O. rebecca*. (**A**) genome-wide estimates of Fst and elevated divergent loci detected in *O. lacepedii*; (**B**) genome-wide estimates of Fst and elevated divergent loci detected in *O. rebecca*. All results are averaged over 100 kb windows and regions of the genome with both Fst estimates and Pi greater than the 95th percentile of the empirical distribution (elevated divergent loci) are marked with red.

Using both metrics of Fst and pairwise nucleotide diversity (Pi), we defined 299 (across 28 scaffolds) and 879 (across 34 scaffolds) windows that displayed signature of selection, containing 588 and 1202 candidate genes under positive selection in *O. lacepedii* and *O.rebecca*, respectively (Figure 6 and Figure 7; Tables S2–S4). These adaptive loci appeared, in general, to be uniformly distributed across the full genomic regions, instead of restricted to narrow “genomic islands” (Figure 7). Functional enrichment (GO) analysis revealed that the top PSGs in *O. lacepedii* were significantly enriched in multiple biological processes, including substance biosynthesis and metabolism, ATP production, cellular component organization, immunity, and DNA metabolic process (Figure S2; Table S5). KEGG enrichment analysis indicated that ‘Glycerophospholipid metabolism (ko00564)’, ‘Glycerolipid metabolism (ko00561)’, ‘Arginine and proline metabolism (ko00330)’, ‘Glutathione metabolism (ko00480)’, and ‘Citrate cycle (ko00020)’ pathways (Figure S3; Table 3) were significantly enriched (*p* < 0.05), which suggested a crucial role of substance metabolism, and energy production in climate adaptation of higher latitude of *O. lacepedii*. While among the candidate genes under strong selection in *O. rebecca*, ‘metabolic pathways (ko01100)’, ‘Glycosphingolipid biosynthesis (ko00604)’, ‘Pyruvate metabolism (ko00620)’, ‘Neuroactive ligand-receptor interaction (ko04080)’, and ‘GnRH signaling pathway (ko04912)’ were significantly enriched (Figures S4 and S5; Table S6 and Table 4), suggesting the involvement of substance metabolism, energy production, environmental information processing, and endocrine system in climate adaptation of lower latitude of *O. rebecca*. Combining both data matrix of these PSGs, 60 genes (Table S4) associated with lipid metabolism (*CMKLR1*, *SIK2*, *ARID5B*, and *VPS13B*) [26,27,28,29], energy production (*CYCS*, and *ES1*) [30,31] and neural development (*BRINP3*, *GRIA3*, *D215*, *SNAP25A*, *KCNN2*, *AMIGO1*, *PRDM13*, *SYN2*, *RBM45*, *OTPB*, and *SHC2*) [32,33,34,35,36,37,38,39,40,41,42] are positively selected in overlap in both *Odontamblyopus* gobies, implying their critical roles in adaptation to contrasting climate conditions expected from the latitude gradient (e.g., the yearly averaged surface water temperature vary from ~10 °C in Dalian to ~25 °C in the Zhongshan sea area, see Figure 1).

## 4. Discussion

### 4.1. High Differentiation between the Two Sister Species

Consistent with the expectation from parapatric speciation, where interspecific differentiation will be accumulated due to the geographic segregation, our whole-genome comparisons of *O. rebecca* and *O. lacepedii* revealed high baseline differentiation between the two species (genome-wide *Fst* = 0.313). Such high divergence could be partly explained by the high density of fixed SNPs (900,408), which appeared to be uniformly distributed across the genome. This pattern of genomic differentiation shared commonalities with that found in the Sebastes rockfish *Sebastes miniatus* and *S. crocotulus*, which exhibited high baseline divergence in parapatry (*Fst* = 0.275), and a large number of fixed SNPs [43]. Conversely, Sebastes rockfish exhibited a slightly deeper divergence time (~2.6 Ma) than the roughly 1.46 Ma estimated for *O. rebecca* and *O. lacepedii* (Figure 5). Our findings also differed from genomic comparisons among other shallower diverged taxa. For instance, in *Anguilla anguilla* and *A. rostrata*, Jacobsen et al. [44] and Pujolar et al. [45] found only a small number of fixed SNPs and elevated regions across the entire genome. The high levels of baseline divergence observed in these two *Odontamblyopus* gobies may provide important resources for understanding on how selective and demographic processes could shape patterns of genetic differentiation in marine teleost.

### 4.2. Demographic Processes in Shaping the Genomic Landscape of the Two Sister Species

Demographic processes, involving a complex history of differentiation, and secondary contact coupled with limited contemporary gene flow, are likely essential factors in driving the high baseline divergence observed between *O. rebecca* and *O. lacepedii*. It was originally hypothesized (based on the mitochondrial DNA) [13] that the ancestral population was isolated by the Pleistocene glaciation, resulting in a split of a northern (*O. lacepedii*) and a southern population (*O. rebecca*). After the ice mass recession, they secondarily contacted at the ocean region around the Taiwan Strait [13]. Such a hypothesis was well supported by the results of our analyses (Figure 2, Figure 3 and Figure 4), except that the divergence time was a little bit later than previously calibrated (1.46 Ma vs. 2.31 Ma) (Figure 5), which falls into a major glaciation period of Donau at the middle Pleistocene [46,47,48]. Given repeating patterns of glaciation and retreat in this region after the time of divergence [47,48], it is possible that the populations expanded and retracted into refugia on more than one occasion, as evidenced by the fluctuation of the effective population size (Ne) in both *O. rebecca* and *O. lacepedii* (Figure 5B). Such a historical fluctuation in population sizes may facilitate random drift via founder events [49], and hence shape the genomic divergence between the two taxa.

In addition to the historical process of glacial isolation, patterns of contemporary gene flow may also reinforce the genomic divergence between the two taxa. Despite sharing a common ancestor, little gene flow was detected after the divergence between *O. rebecca* and *O. lacepedii.* The modeling based on the dadi method preferred an asymmetrical but quite restricted gene flow with a low migration rate (1.20 × 10^−7^, and 4.07 × 10^−9^ for asymmetrical migration) after the divergence between the two taxa (Figure 5B). Such inference of limited gene flow coupled with low migration rate was further supported by results of our classical structure analyses, where no admixture was observed between the two species (Figure 2), suggesting admixture is not common or recent. Such historical demographic processes may have driven the divergence to progress unfettered from the potential homogenizing effects of gene flow [50]. Therefore, under this evolutionary scenario, drift associated with both historical and contemporary processes seemed to play important roles in shaping the high baseline divergence observed in the two *Odontamblyopus* gobies.

### 4.3. Climate Adaptation in Driving Genomic Divergence between the Two Sister Species

Although the divergence observed in the two *Odontamblyopus* gobies can be largely explained by neutral processes, as described above, some outlier regions can also be strongly influenced by natural selection [51]. Consistent with this inference, we identified 299 (across 28 scaffolds) and 879 (across 34 scaffolds) windows that showed selective signals, containing 588 and 1202 candidate genes under strong selection in *O. lacepedii* and *O.rebecca*, respectively (Figure 6 and Figure 7; Tables S2 and S3). This lends support to the role of divergent selection in shaping the observed patterns of genome-wide divergence between the two taxa. However, the adaptive loci appeared to evenly distribute across the genome instead of clustering into narrow “genomic islands” (Figure 7), as observed in multiple pairs of cryptic teleost species that diverged with ongoing gene flow which reflects an incipient stage of speciation [45,52]. Such a pattern of genomic landscape with peaks of elevated divergence widely spreading throughout the genomes is consistent with expectations for populations between *O. lacepedii* and *O.rebecca* where high differentiation has been accumulated. Despite the distinct patterns of genome-wide differentiation, such a scenario of ecological speciation may predict the adaptive merits of these elevated genomic regions underlying adaptations to latitude gradient confronted by *O. lacepedii* and *O.rebecca* populations in parapatry. Indeed, our functional enrichment analysis revealed that the top PSGs in *O. lacepedii* were significantly enriched in pathways associated with substance metabolism, and energy production, whereas the genes selected in *O. rebecca* were primarily enriched in pathways of substance metabolism, energy production, environmental information processing, and the endocrine system (Figures S2–S5; Table 3, Table 4, Tables S5 and S6). The contrasting patterns of natural selection in high- and low-latitude populations may reflect different strategies of species in adaptation to varied local climates they may confront [53]. However, both *O. lacepedii* and *O. rebecca* possessed PSGs enriched in similar pathways of substance metabolism, and energy production may indicate their crucial roles in climate adaptations expected from the latitude gradient (see Figure 1).

Among them, 26 and 34 PSGs associated with mitochondrial genesis, and aerobic respiration, may occur in thermal adaptation function. Two of them (*CSCS*, and *ES1*) were the most compelling candidates, because they exhibited overlapped elevated genomic regions in both *O. lacepedii* and *O. rebecca* (Table S4). Both of the two genes encode either the dominant structural components [31], or central electron carrier proteins that co-ordinates the electron flow among different redox partners that are indispensable for maintaining the normal function of the respiratory chain and hence aerobic energy production in vertebrate mitochondrion [30]. The apparent positive selection in these mitochondrion-associated genes in both species may reflect different energy requirements in adaptation to varied thermal conditions expected from latitude gradient (see Figure 1) [54,55]. Another group of candidate genes likely linked to latitude thermal adaptation were those enriched in pathways of substance metabolism. The most compelling candidates were several lipid metabolic genes (*CMKLR1*, *SIK2*, *ARID5B*, and *VPS13B*) [26,27,28,29] that were positively selected in overlap in both O. *lacepedii* and O. *rebecca* (Table S4). Among them, *CMKLR1* encodes a receptor for chemoattractant adipokine chemerin/RARRES2, and functions through interaction with RARRES2 to initiate activation of G proteins G(i)/G(o) and beta-arrestin pathways leading to multifunctional effects, such as adipogenesis [28]. Whereas *SIK2* encodes a serine/threonine-protein kinase that negatively regulates CREB activity by phosphorylating the CREB-specific coactivator and plays an essential role in fatty acid oxidation and thus fat deposition [26]. The mutations or abnormal expression of *CMKLR1* and *SIK2* would largely result in changed thermogenesis and thermal tolerance in animals [56,57]. Although, the close links between these lipid metabolic genes and the thermal tolerance are less well established in teleosts, the potential contribution of lipid metabolism to their thermal adaptation has also been increasingly documented in several fish taxa [58,59]. Therefore, the apparent positive selection in genes associated with lipid metabolism may indicate their roles in adaptation to contrast thermal conditions expected from the latitude gradient (see Figure 1).

Interestingly, several top PSGs in the two *Odontamblyopus* gobies were significantly enriched in pathways associated with neural development or synaptic function, such as ‘Neuroactive ligand-receptor interaction (ko04080)’ (Table 4). This seems consistent with what has been revealed from the limited literature that neuronal signaling is also involved in the regulation of thermal tolerance in animals [60,61], including fishes [62,63], though the exact mechanism is less known. Udayantha et al. [63] speculated that neuronal signaling may contribute to the thermal adaptation in teleost because thermal stress affects the nervous system, including brain development and function. In addition, the neural system influences behaviors (such as feeding and avoiding), immunity, and reproduction that may affect the fitness and hence adaptation of fish populations under thermal stress [64]. Therefore, the substantial overlapped PSGs associated with neuronal signaling observed in both *O. lacepedii* and *O. rebecca* may indicate their roles in adaptation to contrasting thermal conditions they inhabit.

### 4.4. Climate Adaptation May Underlie the Ecological Speciation of the Two Sister Species

In addition to candidate genes that were in line with the expectation from latitude climate adaptations, several PSGs significantly enriched in pathways associated with puberty, sexual maturation and reproduction, such as ‘GnRH signaling (ko04912)’ and ‘Progesterone -mediated oocyte maturation’ (ko04914) (Figure S5; Table 4), were also identified in both *O. lacepedii* (*SPATA2*, *IZUMO1*, and *VDAC2*) and *O. rebecca* (*GNRHR2*, *ERR3*, *SPATA1*, *ZP3R*, and *SPACA6*) (Tables S2 and S3). Among them, *GNRHR2* encodes a receptor for gonadotropin-releasing hormone II, which promotes puberty and sexual maturation by association with G proteins that activate a phosphatidylinositol-calcium second messenger system [65]. *IZUMO1* and *ZP3R* encode proteins at the cell surface or zona pellucida of oocytes (oolemma), which are essential for species-specific gamete recognition and fertilization in reproduction [66,67]. The significantly positive selection in these reproduction-associated genes may indicate their roles for pre-mating barriers in speciation between the two *Odontamblyopus* gobies. Such reproductive barriers are traditionally recognized as a by-product of divergent adaptation, and may evolve with a hitchhike mechanism through linkage disequilibrium. Under such a by-product speciation scenario, divergent adaptation is thought to come first, and the pre-mating isolation emerges only as a consequence (not as a driver or facilitator) of divergence [7,68]. However, there is accumulating evidence in recent years suggesting that they may emerge simultaneously by endowing these presumed reproduction-related genes with the merit of ecological adaptations. For instance, there are several lines of evidence showing that the originally recognized reproduction-associated gene of gonadotropin-releasing hormone receptors (GnRHRs) may also likely play important roles in thermal adaptations in frog, *Rana luteiventris* [69] and goats [70]. The previously presumed zona pellucida sperm-binding protein (ZP3) associated with sperm–egg interaction has also been implicated in cold tolerance in teleost fish by serving as a glycoprotein [71,72]. Therefore, our results suggested that climate adaptation may directly impact the reproduction-associated genes that contribute to the build-up of the pre-mating barriers instead of evolving as a byproduct that emerged as a consequence of hitchhiking. It is possible that such climate adaptation may have contributed to the built-up of the pre-mating barriers of the split ancestral populations and hence the speciation of the two *Odontamblyopus* gobies. These observations would largely increase our understanding of the role of climate adaptations in ecological speciation and hence the evolution of biodiversity in marine teleosts.

## 5. Conclusions

Our study demonstrated the strengths of comparing the genomes of closely related taxa to shed light on the climate-driven adaptations and their potential roles in generating genetic divergence and ecological speciation of non-model taxa. Using large-scale genomic re-sequencing data, we revealed both genetic drift and divergent selection of climate may function in shaping the high baseline genetic divergence observed between the two *Odontamblyopus* gobies. We also identified several candidate genes that exhibited elevated divergence between the two sister species, including genes associated with substance metabolism, energy production, and response to environmental cues, that may underlie the ecological adaptation expected from the latitude gradient. Some candidate genes linked to the time of puberty and gamete recognition and fusion, also exhibited elevated divergence, possibly indicating their roles in the pre-zygote isolation of the two species in addition to the climate adaptation. The results obtained in this study may not only add to a body of empirical evidence describing underlying mechanisms driving adaptations to latitude gradient, but also expand our knowledge on the genetic mechanisms of how climate-driven adaptations may help generate the genetic divergence, reproductive isolation, and hence speciation between the closely related taxa, such as in marine teleosts.

## Figures and Tables

**Table 1:** The sampling locations and the statistics of genetic diversity in populations of *O. lacepedii* and *O. rebecca*.

Species	Populations	Sample Size	Polymorphic Loci Rate %	Pi	Ho	He
*O. lacepedii*	LNDD 124°11′ E 39°53′ N	15	0.435	0.134	0.135	0.129
	SDQD 120°34′ ’E 36°06′ N	15	0.479	0.136	0.137	0.131
	JSGY 119°13′ E 34°52′ N	16	0.485	0.139	0.141	0.134
	ZJPY 120°38′ E 27°35′ N	15	0.504	0.139	0.142	0.134
	Total	61	0.611	0.142	0.138	0.141
*O. rebecca*	ZJPY 120°38′ E 27°35′ N	12	0.525	0.180	0.182	0.173
	FJLH 118°02′ E 24°26′ N	13	0.532	0.181	0.182	0.174
	GDGZ 113°38′ E 22°27′ N	13	0.535	0.181	0.185	0.174
	Total	38	0.585	0.184	0.183	0.181

Note: LNDD, SDQD, JSGY, ZJPY, FJLH, GDGZ represent samples collected from Dandong (Liaoning province), Qingdao (Shandong province), Ganyu (Jiangsu province), Pingyang (Zhejiang province), Longhai (Fujian province) and Guangzhou (Guangdong province), respectively. Pi, Ho, He represent diversity parameters of nucleotide diversity, heterozygosity observed, and heterozygosity expected.

**Table 2:** Results of dadi analyses based on seven models of demographic scenarios.

Model	No. of Parameters	Log-Likelihood	AIC	Chi-Squared	Theta
AM	5	−2,387,881.23	4,775,772.46	6,366,869.48	2,501,800.09
AMSC	6	−2,812,591.45	5,625,194.90	8,890,476.35	2,178,864.56
SMSC	5	−3,179,893.39	6,359,796.78	7,341,039.78	465,135.90
SM	4	−3,229,862.23	6,459,732.46	7,109,729.31	171,988.45
NM	3	−3,403,932.17	6,807,870.34	21,217,197.42	2,214,644.36
ASM	5	−4,324,433.56	8,648,877.12	24,440,092.25	306,501.13
AAM	6	−4,868,970.52	9,737,953.04	2,439,430,000	280,478.01

Note: AM, AMSC, SMSC, SM, NM, ASM, AAM represent seven demographic scenarios referring to a model of divergence with continuous asymmetric migrations, a model of divergence with asymmetric migrations following secondary contact; a model of divergence with symmetric migrations following secondary contact, a model of divergence with continuous symmetric migrations, a model of divergence with no migrations, a model of divergence with ancient symmetrical migration, a model of divergence with ancient asymmetrical migration, respectively. The model with lowest log-likelihood and AIC value are the best fit model that may reflect the true demographic processes.

**Table 3:** Enrichment of KEGG pathways of positively selected genes in *O. lacepedii* (*p* < 0.05).

KEGG ID	Description	Count	Background Number	*p* Value	P.adjust
ko00561	Glycerolipid metabolism	7	84	1.12 × 10^−2^	6.69 × 10^−1^
ko00330	Arginine and proline metabolism	5	52	1.82 × 10^−2^	6.69 × 10^−1^
ko00564	Glycerophospholipid metabolism	8	116	1.87 × 10^−2^	6.69 × 10^−1^
ko00020	Citrate cycle (TCA cycle)	4	37	2.37 × 10^−2^	6.69 × 10^−1^
ko00480	Glutathione metabolism	5	67	4.40 × 10^−2^	9.41 × 10^−1^

**Table 4:** Enrichment of KEGG pathways of positively selected genes in *O. rebecca* (*p* < 0.05).

KEGG ID	Description	Count	Background Number	*p* Value	P.adjust
ko01100	Metabolic pathways	131	1727	1.97 × 10^−3^	1.83 × 10^−1^
ko04910	Insulin signaling pathway	21	175	2.78 × 10^−3^	1.83 × 10^−1^
ko00604	Glycosphingolipid biosynthesis—ganglio series	6	24	5.26 × 10^−3^	1.83 × 10^−1^
ko04012	ErbB signaling pathway	15	117	6.17 × 10^−3^	1.83 × 10^−1^
ko00601	Glycosphingolipid biosynthesis—lacto and neolacto series	7	35	7.54 × 10^−3^	1.83 × 10^−1^
ko04210	Apoptosis	21	195	8.47 × 10^−3^	1.83 × 10^−1^
ko04912	GnRH signaling pathway	16	134	8.71 × 10^−3^	1.83 × 10^−1^
ko00620	Pyruvate metabolism	8	47	1.01 × 10^−2^	1.86 × 10^−1^
ko04914	Progesterone-mediated oocyte maturation	14	117	1.34 × 10^−2^	2.18 × 10^−1^
ko00270	Cysteine and methionine metabolism	9	63	1.71 × 10^−2^	2.50 × 10^−1^
ko04514	Cell adhesion molecules (CAMs)	21	212	1.87 × 10^−2^	2.50 × 10^−1^
ko04080	Neuroactive ligand-receptor interaction	43	529	2.47 × 10^−2^	2.93 × 10^−1^
ko04371	Apelin signaling pathway	18	180	2.59 × 10^−2^	2.93 × 10^−1^
ko04020	Calcium signaling pathway	27	305	2.86 × 10^−2^	3.00 × 10^−1^
bpec04625	C-type lectin receptor signaling pathway	14	132	3.10 × 10^−2^	3.04 × 10^−1^
bpec04530	Tight junction	24	273	3.97 × 10^−2^	3.65 × 10^−1^
bpec04350	TGF-beta signaling pathway	13	127	4.57 × 10^−2^	3.95 × 10^−1^
bpec00280	Valine, leucine and isoleucine degradation	7	54	4.98 × 10^−2^	3.96 × 10^−1^

## Data Availability

All raw genome re-sequencing data for *O. lacepedii* and *O. rebecca* genome was deposited at the NCBI in the sequence read archive (SRA) under accession number (BioProject Number: PRJNA1006622).

## Supplementary Materials

The following supporting information can be downloaded at: https://www.mdpi.com/article/10.3390/ani13203240/s1.
