# Supplementary material for: Climate Adaptation and Drift Shape the Genomes of Two Eel-Goby Sister Species Endemic to Contrasting Latitude
Source: Animals (Basel). 2023 Oct 17;13(20):3240. doi: 10.3390/ani13203240 (PMC10603712; doi:10.3390/ani13203240)
Supplement: Supplementary file 1 [file animals-13-03240-s001.zip › Supplementary Tables.pdf]

Table S1. Statistics of genome re-sequencing data for *O. lacepedii* and *O. rebecca* produced by MGIDNB T7 platform.

Species	Clean reads	Mapped reads	Mapping rate	Average depth	Coverage4X	SNPs
O. lacepedii	10133065798	10069161829	99.37%	26.88	87.77%	6373895
O. rebecca	6092856242	6034641939	99.01%	26.75	92.62%	7190587
Total	16225922040	16103803768	99.23%	26.83	90.76%	9972482

Table S2. Candidate positively selected genes in *O. lacepedii* ($p < 0.05$).

Gene ID	Swissprot Annotation	Gene ID	Swissprot Annotation	Gene ID	Swissprot Annotation
Ola0026430.1	*DLG4*	Ola0227690.1	*MKNK1*	Ola0104220.1	*SORT1*
Ola0227710.1	*MBX1*	Ola0119320.1	*MKS3*	Ola0188520.1	*SPAT2*
Ola0228420.1	*DMRTA2*	Ola0181830.1	*MN1*	Ola0099910.1	*SPDEF*
Ola0222200.1	*DNAJB5*	Ola0077880.1	*MNX1*	Ola0209360.1	*SPIR1*
Ola0112990.1	*DNJB9*	Ola0227680.1	*MOB3C*	Ola0228410.1	*SPRR1A*
Ola0113000.1	*DNM1L*	Ola0073310.1	*MORN3*	Ola0038560.1	*SPRE1*
Ola0130730.1	*DOK3*	Ola0165340.1	*MOT10*	Ola0214680.1	*SPRY7*
Ola0074110.1	*DOPO*	Ola0221330.1	*MSH3*	Ola0048780.1	*SRCA*
Ola0228280.1	*DPOE4*	Ola0059860.1	*MTMRA*	Ola0092030.1	*SRPK1*
Ola0058170.1	*DPOLN*	Ola0074190.1	*MUT7*	Ola0222270.1	*SRR1*
Ola0040780.1	*D215*	Ola0214950.1	*MYCB2*	Ola0073040.1	*SRSF9*
Ola0073720.1	*DRG1*	Ola0059850.1	*MYO1E*	Ola0182580.1	*SSH1*
Ola0186470.1	*DS13A*	Ola0221320.1	*MYORG*	Ola0038540.1	*ST14*
Ola0059260.1	*DS22A*	Ola0059760.1	*MYZAP*	Ola0073710.1	*STAR7*
Ola0188160.1	*DUS19*	Ola0070130.1	*NAA25*	Ola0028640.1	*STC2*
Ola0059470.1	*E2F5*	Ola0074180.1	*NARPA*	Ola0194050.1	*STK11*
Ola0209390.1	*ADNP2*	Ola0188150.1	*NCKP1*	Ola0077890.1	*STK16*
Ola0228260.1	*EAA1*	Ola0188600.1	*NDST2*	Ola0026100.1	*STK36*
Ola0130890.1	*EDA*	Ola0194020.1	*NDUAD*	Ola0059460.1	*STRC*
Ola0111250.1	*EDC4*	Ola0059700.1	*NEDD4L*	Ola0186830.1	*SUFU*
Ola0074240.1	*EDEM3*	Ola0188620.1	*NETR*	Ola0194060.1	*SUGP1*
Ola0026450.1	*ADT2*	Ola0130790.1	*NF7O*	Ola0058220.1	*SVEP1*
Ola0228050.1	*AES*	Ola0222240.1	*NFH*	Ola0182600.1	*SVOP*
Ola0038590.1	*EHD2*	Ola0104460.1	*NFYA*	Ola0082320.1	*SOWAHB*
Ola0117130.1	*EIF3H*	Ola0104470.1	*NGRN*	Ola0074130.1	*SWI5*
Ola0227670.1	*ELOV1*	Ola0228290.1	*NIM1*	Ola0072940.1	*BMR1B*
Ola0059240.1	*EMC8*	Ola0214030.1	*NNRD*	Ola0181420.1	*SYN2*
Ola0222190.1	*EPG5*	Ola0222210.1	*A1A1A*	Ola0038650.1	*SYNG1*
Ola0026420.1	*EPHB4*	Ola0186840.1	*NO29*	Ola0104210.1	*SYPL2*
Ola0186160.1	*AGRB3*	Ola0186450.1	*NPY1R*	Ola0061330.1	*SYT11*
Ola0185990.1	*ERLEC*	Ola0130780.1	*NPY2R*	Ola0073300.1	*T120B*
Ola0214980.1	*ES1*	Ola0214740.1	*NR0B1*	Ola0165380.1	*TAB2*
Ola0117110.1	*EXT1B*	Ola0185980.1	*NR1AA*	Ola0113190.1	*TAF3*

Ola0186170.1	*EYS*	Ola0105170.1	*NR1D2*	Ola0070120.1	*BRAP*
Ola0073670.1	*F136A*	Ola0059540.1	*NRN1L*	Ola0118180.1	*TBC15*
Ola0130750.1	*F193B*	Ola0221200.1	*NSD3*	Ola0195110.1	*BRINP3*
Ola0113180.1	*F208B*	Ola0117090.1	*NSE2*	Ola0088010.1	*BRSK2*
Ola0182300.1	*F219A*	Ola0111260.1	*NTF2*	Ola0222220.1	*TERA*
Ola0061360.1	*F219B*	Ola0105660.1	*NTF7*	Ola0119290.1	*TEX10*
Ola0111430.1	*FA2H*	Ola0049360.1	*NTH*	Ola0059720.1	*TEX9*
Ola0228150.1	*FA32A*	Ola0105970.1	*NUAK1*	Ola0048790.1	*TFAP4*
Ola0229430.1	*FA43A*	Ola0038580.1	*NUP88*	Ola0232810.1	*TFE3*
Ola0209430.1	*FA83H*	Ola0188560.1	*NVL*	Ola0222260.1	*TFP11*
Ola0117080.1	*FA84B*	Ola0204880.1	*O52D1*	Ola0195640.1	*TFR1*
Ola0228430.1	*FAF1*	Ola0227950.1	*OD3L2*	Ola0078010.1	*TGFA1*
Ola0221280.1	*FAHD2*	Ola0186850.1	*OGA*	Ola0082330.1	*THAP1*
Ola0222230.1	*FANCG*	Ola0182550.1	*OMGP*	Ola0227650.1	*BT3L4*
Ola0116240.1	*FAXC*	Ola0229470.1	*OPA1*	Ola0118120.1	*BTG3*
Ola0227720.1	*FBN1*	Ola0038670.1	*OPA3*	Ola0058200.1	*THIO*
Ola0188580.1	*FBX28*	Ola0232790.1	*OPSB*	Ola0222250.1	*THOC5*
Ola0077990.1	*FBXL3*	Ola0104550.1	*ASB8*	Ola0110690.1	*TIGRB*
Ola0116230.1	*FBXL4*	Ola0028620.1	*OPSP*	Ola0227870.1	*TIKI2*
Ola0223770.1	*FCSD1*	Ola0095310.1	*OSR2*	Ola0228040.1	*TLE1*
Ola0181430.1	*AJM1*	Ola0221370.1	*OTPB*	Ola0060340.1	*TLN2*
Ola0209420.1	*AKIB1*	Ola0182590.1	*OXDA*	Ola0186420.1	*TM14A*
Ola0110700.1	*FGF23*	Ola0049580.1	*P5CR1*	Ola0182270.1	*TMEM2*
Ola0110710.1	*FGF6*	Ola0194010.1	*P66A*	Ola0187910.1	*TMM26*
Ola0182520.1	*FICD*	Ola0070150.1	*PA2G3*	Ola0229460.1	*TMM44*
Ola0118550.1	*FKB14*	Ola0227850.1	*PACE1*	Ola0195610.1	*TMM8A*
Ola0059780.1	*AL1A2*	Ola0099920.1	*PACN1*	Ola0187930.1	*TNAP3*
Ola0086430.1	*FMN2*	Ola0111380.1	*PAG15*	Ola0113040.1	*TNNI3*
Ola0227900.1	*FOXD2*	Ola0072460.1	*PAPP1*	Ola0113010.1	*TNNT2*
Ola0227910.1	*FOXE4*	Ola0181450.1	*PBX3*	Ola0070110.1	*TOR4A*
Ola0059280.1	*FOXF1*	Ola0221260.1	*PCNA*	Ola0228220.1	*TPM1*
Ola0228120.1	*FUMH*	Ola0077900.1	*PCNP*	Ola0207440.1	*TPPC9*
Ola0059450.1	*FURI1*	Ola0049540.1	*PCY2*	Ola0028610.1	*TPST1*
Ola0227740.1	*FYCO1*	Ola0073680.1	*PCYOX*	Ola0214690.1	*TRI13*
Ola0073060.1	*G3ST1*	Ola0072960.1	*PDLI5*	Ola0072440.1	*TRI32*
Ola0113090.1	*GALR2*	Ola0119330.1	*PDP1*	Ola0186820.1	*TRIM8*
Ola0181440.1	*GAPD1*	Ola0187920.1	*PERP*	Ola0228080.1	*C2C4C*
Ola0197030.1	*GAT6A*	Ola0104530.1	*PFKAM*	Ola0182280.1	*TRPM3*
Ola0133670.1	*GCR*	Ola0214650.1	*PHF6*	Ola0049350.1	*TSC2*
Ola0209400.1	*GGCT*	Ola0026120.1	*PHLDB1*	Ola0105670.1	*TSN2*
Ola0227730.1	*GHRHR*	Ola0059900.1	*PHLP2*	Ola0116280.1	*TSTD3*
Ola0073020.1	*GIT2*	Ola0215550.1	*PHOCN*	Ola0181810.1	*TTC28*
Ola0074160.1	*AMBP*	Ola0181820.1	*PIPNB*	Ola0227840.1	*CA112*
Ola0104190.1	*AMIGO1*	Ola0181620.1	*PIWL1*	Ola0228250.1	*TX1B3*

Ola0228070.1	*GNA11*	Ola0049340.1	*PKD1*	Ola0228100.1	*UB2R1*
Ola0104170.1	*GNAI3*	Ola0118540.1	*PKHA8*	Ola0214050.1	*UBE2A*
Ola0049530.1	*GNAO*	Ola0186430.1	*PLCE*	Ola0073090.1	*CABP7*
Ola0228060.1	*GNAQ*	Ola0072450.1	*ASTN2*	Ola0105190.1	*CACO1*
Ola0232800.1	*GNL3L*	Ola0112980.1	*PLPL8*	Ola0110670.1	*CAD13*
Ola0221290.1	*GPAT2*	Ola0221190.1	*PLPP4*	Ola0119340.1	*CAD17*
Ola0104180.1	*GPR61*	Ola0214630.1	*AT11A*	Ola0116270.1	*UBP45*
Ola0186000.1	*GPR75*	Ola0229450.1	*AT133*	Ola0130710.1	*UNC5A*
Ola0130900.1	*GRIA3*	Ola0113030.1	*PMGE*	Ola0072930.1	*UNC5C*
Ola0094900.1	*GRIK5*	Ola0116260.1	*PNISR*	Ola0118150.1	*UPAR*
Ola0059770.1	*GRL1A*	Ola0104200.1	*AT7L2*	Ola0073260.1	*CAH15*
Ola0038690.1	*GRP1*	Ola0026110.1	*ATF5*	Ola0111350.1	*CAH5B*
Ola0059220.1	*GSE1*	Ola0104560.1	*ATF7*	Ola0111390.1	*USB1*
Ola0094920.1	*GSK3B*	Ola0059480.1	*PPGB*	Ola0222130.1	*UT2*
Ola0186410.1	*GSTA3*	Ola0165390.1	*PPIL4*	Ola0059510.1	*CALB2*
Ola0078000.1	*GSTT1*	Ola0074170.1	*PPN1*	Ola0113020.1	*CALD1*
Ola0026520.1	*GSX1*	Ola0059200.1	*PPR3*	Ola0070100.1	*VDAC2*
Ola0074090.1	*GT253*	Ola0186860.1	*PPRC1*	Ola0116290.1	*VIM*
Ola0073740.1	*GTR5*	Ola0116300.1	*PRDM13*	Ola0095300.1	*VPS13B*
Ola0118520.1	*HACL1*	Ola0130770.1	*PRLD1*	Ola0209460.1	*VPS28*
Ola0058180.1	*HAUS3*	Ola0073050.1	*PROD*	Ola0209380.1	*VTC1A*
Ola0227920.1	*HAUS8*	Ola0195100.1	*PRP2*	Ola0215380.1	*VWA8*
Ola0232780.1	*HCFC1*	Ola0088000.1	*PRR12*	Ola0117100.1	*WASC5*
Ola0194070.1	*HCN2*	Ola0059690.1	*PRTGA*	Ola0026530.1	*WASF3*
Ola0228110.1	*HDGR2*	Ola0059190.1	*ATMIN*	Ola0186020.1	*WDR27*
Ola0214060.1	*ANR10*	Ola0130720.1	*ATOH1*	Ola0060460.1	*WDR76*
Ola0078500.1	*ANR11*	Ola0059230.1	*PSF2*	Ola0082350.1	*WDR91*
Ola0118510.1	*ANR28*	Ola0186010.1	*PSM4A*	Ola0182500.1	*WSCD2*
Ola0215590.1	*ANR44*	Ola0038570.1	*PSMD8*	Ola0229420.1	*XXLT1*
Ola0099880.1	*ANS1A*	Ola0074150.1	*CANLF*	Ola0194040.1	*CBARP*
Ola0074230.1	*ANXA3*	Ola0058190.1	*PTGR1*	Ola0048760.1	*CBP*
Ola0059890.1	*AP1G1*	Ola0095190.1	*PTN2*	Ola0221270.1	*YH24*
Ola0038680.1	*HNRL1*	Ola0073070.1	*QCR9*	Ola0113050.1	*YOW5*
Ola0228090.1	*AP3D1*	Ola0119310.1	*R12BB*	Ola0077940.1	*CCD81*
Ola0228240.1	*HSH2D*	Ola0223790.1	*RGS9BP*	Ola0059730.1	*Z280D*
Ola0104440.1	*HSP70*	Ola0214020.1	*RAB20*	Ola0026440.1	*ZBT20*
Ola0059740.1	*HTF4*	Ola0232770.1	*RAB7*	Ola0188170.1	*ZC3HF*
Ola0028630.1	*HTSF1*	Ola0228230.1	*RAB8A*	Ola0026540.1	*ZDH23*
Ola0105260.1	*HOXC6A*	Ola0117120.1	*RAD21*	Ola0053230.1	*ZDHHC5*
Ola0105250.1	*HOXC8A*	Ola0105180.1	*RARGA*	Ola0026560.1	*ZFX*
Ola0105240.1	*HXC9*	Ola0221230.1	*RASF2*	Ola0227660.1	*ZFYV9*
Ola0105230.1	*HXCAA*	Ola0188180.1	*RBM45*	Ola0204900.1	*ZHX1*
Ola0105220.1	*HXCBA*	Ola0111270.1	*RBP10*	Ola0204890.1	*ZHX2*
Ola0105210.1	*HXCCA*	Ola0214660.1	*RCBT1*	Ola0073080.1	*ZMAT5*

Ola0105200.1	*HXCDA*	Ola0074220.1	*RCL1*	Ola0111400.1	*ZN319*
Ola0077910.1	*I18RA*	Ola0060480.1	*RDH11*	Ola0094910.1	*ZN574*
Ola0038660.1	*IASPP*	Ola0214850.1	*RENR*	Ola0060500.1	*ZN710*
Ola0095210.1	*ID4*	Ola0059710.1	*RFX7*	Ola0059880.1	*ZN821*
Ola0073250.1	*IDD*	Ola0215370.1	*RGCC*	Ola0082310.1	*SLC30A5*
Ola0060490.1	*IDHP*	Ola0060470.1	*RGRF1*	Ola0188610.1	*ZSWM8*
Ola0221220.1	*IF2*	Ola0195070.1	*RGS13*	Ola0110680.1	*CCND2*
Ola0077920.1	*IL18R*	Ola0195090.1	*RGS18*	Ola0059680.1	*CCPG1*
Ola0077930.1	*IL1BP*	Ola0195080.1	*RGS21*	Ola0074200.1	*CD37L*
Ola0077970.1	*ILDR1*	Ola0195620.1	*RGS9*	Ola0221180.1	*CDC45*
Ola0077980.1	*ILDR2*	Ola0026510.1	*RHG31*	Ola0195050.1	*CDC73*
Ola0214670.1	*IMA4*	Ola0073290.1	*RHOF*	Ola0165370.1	*CDK19*
Ola0104230.1	*INAVA*	Ola0087980.1	*RIC8A*	Ola0222110.1	*CDK7*
Ola0214040.1	*ING1*	Ola0181630.1	*RIMB2*	Ola0228440.1	*CDN2A*
Ola0119280.1	*INVS*	Ola0104480.1	*RL10A*	Ola0188550.1	*CDT1*
Ola0087990.1	*IRF3*	Ola0059500.1	*AATM*	Ola0059170.1	*CDYL2*
Ola0059270.1	*IRF8*	Ola0228160.1	*RM54*	Ola0095170.1	*CE192*
Ola0214990.1	*ACOD1*	Ola0059840.1	*RN111*	Ola0182310.1	*CEL2A*
Ola0213990.1	*IRS2B*	Ola0228450.1	*RNF11*	Ola0113200.1	*CELF2*
Ola0182540.1	*ISCU*	Ola0073340.1	*RNF34*	Ola0165400.1	*CENPE*
Ola0188540.1	*APT*	Ola0214710.1	*RNH2B*	Ola0095200.1	*CEP76*
Ola0221310.1	*IZUM1*	Ola0195060.1	*RO60*	Ola0099900.1	*CF106*
Ola0118590.1	*JAZF1*	Ola0188590.1	*RPAC1*	Ola0195630.1	*CG026*
Ola0074210.1	*KAD3*	Ola0028600.1	*CRCP*	Ola0059750.1	*CGNL1*
Ola0214910.1	*KBTB7*	Ola0165350.1	*RPF2*	Ola0215560.1	*CH10*
Ola0105960.1	*KBTBC*	Ola0026090.1	*ATS15*	Ola0215570.1	*CH60*
Ola0215000.1	*KCD12*	Ola0059870.1	*ATX1L*	Ola0118560.1	*CHIO*
Ola0133660.1	*KCD16*	Ola0209370.1	*BAALC*	Ola0073100.1	*CHP3*
Ola0113070.1	*KCNA1*	Ola0214860.1	*BCOR*	Ola0094940.1	*CIC*
Ola0113080.1	*KCNA1*	Ola0099890.1	*RU1C*	Ola0194030.1	*CIRBA*
Ola0105650.1	*KCNA3*	Ola0227810.1	*RXRGB*	Ola0227960.1	*CKS2*
Ola0104430.1	*KCNC4*	Ola0038700.1	*RYR1*	Ola0195650.1	*ACK1*
Ola0118130.1	*KCNN2*	Ola0086450.1	*RYR2*	Ola0214970.1	*CLN5*
Ola0095320.1	*KCNS2*	Ola0073700.1	*S20AB*	Ola0222280.1	*CLU*
Ola0073330.1	*KDM2B*	Ola0221240.1	*S23A2*	Ola0214720.1	*CM042*
Ola0092050.1	*KI21B*	Ola0105950.1	*S26A6*	Ola0182510.1	*CMKLR1*
Ola0188530.1	*KITM*	Ola0074140.1	*S27A4*	Ola0059520.1	*CMTR2*
Ola0182290.1	*KLF9*	Ola0059490.1	*S38A7*	Ola0113060.1	*CNOT4*
Ola0117070.1	*KLH38*	Ola0059530.1	*S38A8*	Ola0214000.1	*CO4A1*
Ola0104540.1	*LARP4*	Ola0110720.1	*S45A3*	Ola0214010.1	*CO4A2*
Ola0111340.1	*LAT1*	Ola0227930.1	*SAC31*	Ola0086440.1	*ACM3*
Ola0053220.1	*SLC43A1*	Ola0111360.1	*SAE2*	Ola0118530.1	*COLQ*
Ola0111370.1	*LCAT*	Ola0228180.1	*SAFB1*	Ola0116250.1	*COQ3*
Ola0186870.1	*LDB1*	Ola0104450.1	*AHCYL2*	Ola0182570.1	*COR1C*

Ola0221210.1	*LETM2*	Ola0186460.1	*SAMD8*	Ola0059250.1	*COX41*
Ola0209440.1	*GRINA*	Ola0105980.1	*SAMH1*	Ola0061370.1	*COX5A*
Ola0092040.1	*LHPL5*	Ola0074100.1	*SARDH*	Ola0059180.1	*COXM2*
Ola0228200.1	*LIPL*	Ola0182530.1	*SART3*	Ola0049560.1	*CP110*
Ola0026550.1	*LCN1*	Ola0105940.1	*SC6A8*	Ola0227940.1	*CPAMD8*
Ola0215010.1	*LMO7*	Ola0221380.1	*SCAMP1*	Ola0088020.1	*CPT1A*
Ola0227820.1	*LMX1A*	Ola0214940.1	*SCEL*	Ola0118570.1	*CPVL*
Ola0227860.1	*LPXN*	Ola0040790.1	*SCN3B*	Ola0073320.1	*CRCM1*
Ola0073350.1	*LRRC75A*	Ola0187940.1	*SDCG8*	Ola0118580.1	*CREB5*
Ola0095160.1	*LRAD4*	Ola0073270.1	*SE1BA*	Ola0028660.1	*CRERF*
Ola0130880.1	*LRC32*	Ola0028650.1	*SEC20*	Ola0214870.1	*CRLA*
Ola0229440.1	*LSG1*	Ola0095180.1	*SEH1*	Ola0059920.1	*CRLD2*
Ola0195600.1	*LURA1*	Ola0061340.1	*SEM7A*	Ola0221250.1	*KCNIP3*
Ola0227830.1	*LYAM1*	Ola0222100.1	*SERC5*	Ola0111410.1	*CSK22*
Ola0214880.1	*M1I1B*	Ola0222120.1	*SETBP*	Ola0227630.1	*CTL5*
Ola0130760.1	*MAD3*	Ola0038600.1	*BICRA*	Ola0214730.1	*CX021*
Ola0049570.1	*MAFG*	Ola0215580.1	*SF3B1*	Ola0130860.1	*CXA3*
Ola0118170.1	*MAG*	Ola0073030.1	*SGSM1*	Ola0130870.1	*CXB1*
Ola0186400.1	*MAK*	Ola0133650.1	*SH3R2*	Ola0232820.1	*CXXC1*
Ola0059910.1	*MALD3*	Ola0227970.1	*SHC2*	Ola0188190.1	*CYCS*
Ola0228170.1	*MALT1*	Ola0227640.1	*SIA7E*	Ola0165360.1	*DCAM*
Ola0048800.1	*MB12A*	Ola0214070.1	*SIK2*	Ola0222290.1	*DDHD2*
Ola0232830.1	*MBD1*	Ola0049550.1	*SIR7*	Ola0060330.1	*DDX28*
Ola0214640.1	*MCF2L*	Ola0214930.1	*SLAI1*	Ola0130740.1	*DDX41*
Ola0186440.1	*MCPH1*	Ola0059830.1	*SLTM*	Ola0188570.1	*DEGS1*
Ola0113170.1	*ARHG8*	Ola0048750.1	*SLX4*	Ola0059810.1	*ADA10*
Ola0061350.1	*ARI3B*	Ola0209410.1	*SMD1*	Ola0221300.1	*ADRA2B*
Ola0187900.1	*ARID5B*	Ola0118140.1	*SMG9*	Ola0215390.1	*DGKH*
Ola0197020.1	*MIB1*	Ola0118160.1	*SMP*	Ola0038550.1	*DHX34*
Ola0059820.1	*MINY2*	Ola0061380.1	*SNAP25A*	Ola0104570.1	*DI2BA*
Ola0209450.1	*MK15*	Ola0026080.1	*SNX19*	Ola0048770.1	*ADCY9*

Table S3. Candidate positively selected genes in *O. rebecca* ($p < 0.05$).

Gene ID	Swissprot Annotation	Gene ID	Swissprot Annotation	Gene ID	Swissprot Annotation
Ola0040110.1	*DJC30*	Ola0117390.1	*SOX4*	Ola0186950.1	*MRP2*
Ola0072090.1	*DJC21*	Ola0068160.1	*SOCS2*	Ola0025950.1	*MPU1*
Ola0166680.1	*ADCY8*	Ola0228510.1	*SOAT1*	Ola0079780.1	*MPSF*
Ola0229220.1	*DIAC*	Ola0061560.1	*SO3A1*	Ola0227190.1	*MP2K2*
Ola0099680.1	*ADCL3*	Ola0116800.1	*SIGLEC1*	Ola0025720.1	*MP17L*
Ola0106080.1	*DHX35*	Ola0073640.1	*SNX24*	Ola0104390.1	*MOT5*
Ola0071770.1	*DHX29*	Ola0040230.1	*SNRPA*	Ola0086650.1	*MOT4*
Ola0099670.1	*DHRS3*	Ola0069210.1	*SNF5*	Ola0187540.1	*MOT12*
Ola0182010.1	*ADB4C*	Ola0188230.1	*SNAB*	Ola0082810.1	*MOC2B*
Ola0226580.1	*DHB7*	Ola0061380.1	*SNAP25A*	Ola0070840.1	*MOB1B*
Ola0025910.1	*DHB12*	Ola0079310.1	*SMYD3*	Ola0028440.1	*MNT*
Ola0028990.1	*ADAD2*	Ola0104830.1	*SMC1A*	Ola0231990.1	*MMP24*
Ola0078880.1	*DGLA*	Ola0072140.1	*SMAD7*	Ola0196290.1	*MMP14*
Ola0025820.1	*DGAT2*	Ola0040330.1	*SIX4*	Ola0069200.1	*MMP11*
Ola0069600.1	*ADRA2B*	Ola0040700.1	*SIK2*	Ola0231970.1	*MLP3A*
Ola0068810.1	*DEN1A*	Ola0071600.1	*SIA8E*	Ola0180760.1	*MLEC*
Ola0059320.1	*DEFI8*	Ola0071350.1	*SIA7F*	Ola0071950.1	*MK10*
Ola0072780.1	*DDX54*	Ola0071360.1	*SIA7C*	Ola0186730.1	*MK08B*
Ola0105900.1	*DDX20*	Ola0040300.1	*SI1L3*	Ola0166650.1	*MIXL1*
Ola0195140.1	*DDR2*	Ola0026190.1	*SI1L2*	Ola0227280.1	*MIS*
Ola0060140.1	*DDIAS*	Ola0227990.1	*SHD*	Ola0079590.1	*MIS12*
Ola0104980.1	*ACVL1*	Ola0072190.1	*SHC3*	Ola0107700.1	*MINT*
Ola0104990.1	*ACV1B*	Ola0227970.1	*SHC2*	Ola0071370.1	*MIGA2*
Ola0070850.1	*DCK*	Ola0040120.1	*SH2B2*	Ola0228010.1	*MIER2*
Ola0117370.1	*DCDC2*	Ola0070980.1	*SGTB*	Ola0195170.1	*MIER1*
Ola0070910.1	*DAB2*	Ola0027170.1	*SG494*	Ola0060080.1	*MICA2*
Ola0078470.1	*ACSF3*	Ola0127810.1	*SFXN1*	Ola0187060.1	*MFRN2*
Ola0061000.1	*CYLD*	Ola0127800.1	*SFR1*	Ola0229130.1	*MFN1*
Ola0188190.1	*CYCS*	Ola0069120.1	*SET*	Ola0073750.1	*5NTC*
Ola0059950.1	*CYB5*	Ola0040960.1	*BICL1*	Ola0028590.1	*ARLY*
Ola0059350.1	*CY24A*	Ola0098740.1	*BICD2*	Ola0073600.1	*METK2*
Ola0126030.1	*CXD2*	Ola0104890.1	*BI51A*	Ola0078640.1	*MET15*
Ola0188200.1	*CXA5*	Ola0041000.1	*SERB*	Ola0061430.1	*MESP2*
Ola0070400.1	*CWC27*	Ola0069690.1	*SEPT5*	Ola0061440.1	*MESP1*
Ola0025650.1	*CWC15*	Ola0028430.1	*SEPT4*	Ola0099300.1	*MEP50*
Ola0040140.1	*CUX1*	Ola0195010.1	*SENP5*	Ola0005420.1	*MEOX2*
Ola0186930.1	*CUTC*	Ola0092830.1	*SEM6D*	Ola0068630.1	*MEG10*
Ola0068660.1	*CTXN3*	Ola0227490.1	*SEM6B*	Ola0069660.1	*MEF2C*
Ola0130590.1	*CTR2*	Ola0072200.1	*SEM4D*	Ola0226830.1	*MEF2B*
Ola0072150.1	*CTIF*	Ola0232920.1	*SEM3F*	Ola0061590.1	*MEF2A*
Ola0133970.1	*CTBP2*	Ola0193390.1	*SELT*	Ola0182030.1	*MED22*

Ola0069630.1	*CSPG2*	Ola0028560.1	*SEBOX*	Ola0099140.1	*MED20*
Ola0117140.1	*CSMD3*	Ola0069760.1	*SE6L1*	Ola0193440.1	*ARL14*
Ola0079770.1	*CSMD1*	Ola0078850.1	*SDHF2*	Ola0070940.1	*ARID5B*
Ola0061300.1	*CSK*	Ola0116780.1	*SDHA*	Ola0152410.1	*ARHL2*
Ola0118340.1	*CSK2B*	Ola0028190.1	*SDF2*	Ola0116990.1	*MDHM*
Ola0073480.1	*KCNIP3*	Ola0071880.1	*SCRB2*	Ola0060260.1	*MDGA1*
Ola0040220.1	*CS054*	Ola0069800.1	*SCRB1*	Ola0104960.1	*ARHGP*
Ola0040280.1	*CS047*	Ola0079340.1	*SCPDL*	Ola0040880.1	*ARHGC*
Ola0195020.1	*CRUM1*	Ola0068180.1	*SCO2*	Ola0107710.1	*MD2L2*
Ola0188250.1	*CRLS1*	Ola0193410.1	*SCHI1*	Ola0130530.1	*MD19A*
Ola0227340.1	*CRLF1*	Ola0070610.1	*SCAMP1*	Ola0072750.1	*MD13L*
Ola0196200.1	*CRJ1C*	Ola0069180.1	*SCAI*	Ola0061580.1	*MCTP2*
Ola0070550.1	*CRHBP*	Ola0025680.1	*SC6A7*	Ola0069680.1	*MCTP1*
Ola0069720.1	*CRBB1*	Ola0071540.1	*SC16A*	Ola0188260.1	*MCM8*
Ola0069730.1	*CRBA4*	Ola0038880.1	*SBDS*	Ola0229170.1	*MCLN2*
Ola0068150.1	*CRADD*	Ola0025990.1	*SAT2*	Ola0060570.1	*MCL1*
Ola0071480.1	*CRA1B*	Ola0073610.1	*SAP*	Ola0086740.1	*MBTD1*
Ola0227100.1	*CR3LB*	Ola0229280.1	*SAM13*	Ola0039830.1	*MBNL1*
Ola0072120.1	*CR032*	Ola0196280.1	*SALL2*	Ola0069170.1	*MB12B*
Ola0071630.1	*CR025*	Ola0060990.1	*SALL1*	Ola0070760.1	*MAST4*
Ola0025850.1	*CPTP*	Ola0099180.1	*AHCYL2*	Ola0229960.1	*MAST3*
Ola0078860.1	*CPSF7*	Ola0118110.1	*SACA6*	Ola0068680.1	*MARH3*
Ola0059970.1	*CPNE7*	Ola0104420.1	*S6A17*	Ola0067180.1	*MAP2*
Ola0227940.1	*CPAMD8*	Ola0098280.1	*S6A13*	Ola0071230.1	*MAP1B*
Ola0072010.1	*CPLX4*	Ola0069780.1	*S4A5*	Ola0116310.1	*MAOX*
Ola0061280.1	*CPLX3*	Ola0070860.1	*S4A4*	Ola0204550.1	*MALD2*
Ola0029000.1	*CPEB4*	Ola0027220.1	*S47A1*	Ola0099440.1	*MAGI3*
Ola0118380.1	*CP21A*	Ola0068470.1	*S38A9*	Ola0079960.1	*MAF*
Ola0186920.1	*COX15*	Ola0195160.1	*S35D2*	Ola0106090.1	*MAFB*
Ola0061570.1	*COT2*	Ola0227370.1	*S35A3*	Ola0116560.1	*MACF1*
Ola0040160.1	*COQA1*	Ola0187980.1	*S29A1*	Ola0000840.1	*MA2A1*
Ola0166700.1	*COLI2*	Ola0068640.1	*S27A6*	Ola0040290.1	*M3K10*
Ola0193450.1	*COLE*	Ola0226850.1	*S2542*	Ola0222410.1	*LZTR1*
Ola0129800.1	*COF2*	Ola0025960.1	*S2535*	Ola0073630.1	*LYOX*
Ola0117230.1	*COBL*	Ola0026840.1	*S22A6*	Ola0068900.1	*LRSM1*
Ola0226410.1	*COA7*	Ola0181400.1	*S20AA*	Ola0071430.1	*LRC8A*
Ola0070900.1	*CO9*	Ola0228740.1	*S1PR4*	Ola0226440.1	*LRC52*
Ola0079320.1	*CNST*	Ola0228000.1	*S1PR3*	Ola0061220.1	*LRC49*
Ola0067920.1	*CNOT2*	Ola0025900.1	*S12A9*	Ola0099660.1	*LRC38*
Ola0187020.1	*CNNM1*	Ola0068700.1	*S12A2*	Ola0061390.1	*LRC28*
Ola0025760.1	*CNKR2*	Ola0070540.1	*S100Z*	Ola0180800.1	*LRRC75A*
Ola0229100.1	*ACL6A*	Ola0026720.1	*RYR1*	Ola0060120.1	*LPIN1*
Ola0068170.1	*CNDH2*	Ola0072020.1	*RX3*	Ola0187090.1	*LOXL4*
Ola0118090.1	*CMKLR1*	Ola0067150.1	*RUXF*	Ola0071610.1	*LOXH1*

Ola0040350.1	*CLPT1*	Ola0187250.1	*ABCGK*	Ola0127780.1	*LOX5*
Ola0130570.1	*CLP1*	Ola0026170.1	*ABCG4*	Ola0068770.1	*LMX1B*
Ola0038860.1	*CLDY*	Ola0188210.1	*RUNX1*	Ola0068920.1	*LMO41*
Ola0038870.1	*CLD6*	Ola0079620.1	*RUFY2*	Ola0068670.1	*LMNB1*
Ola0069700.1	*CLD5*	Ola0070930.1	*RTKN*	Ola0072000.1	*LMAN1*
Ola0038800.1	*CLD4*	Ola0068000.1	*RTCB*	Ola0028450.1	*LIS1B*
Ola0038840.1	*CLD3*	Ola0026010.1	*RT31*	Ola0166660.1	*LIN9*
Ola0222420.1	*CLD22*	Ola0116980.1	*RSPO3*	Ola0078610.1	*LIN7C*
Ola0025980.1	*CLD15*	Ola0116490.1	*RSPO2*	Ola0041020.1	*LIMK1*
Ola0130510.1	*CLCF1*	Ola0070920.1	*BDP1*	Ola0227240.1	*LIGO3*
Ola0229160.1	*CLCC1*	Ola0078620.1	*BDNF*	Ola0000330.1	*LIAT1*
Ola0060050.1	*CLC3A*	Ola0133590.1	*ABCB7*	Ola0070440.1	*LHX5*
Ola0040250.1	*CLASR*	Ola0041890.1	*BC7BB*	Ola0070620.1	*LHPL2*
Ola0059400.1	*CL18A*	Ola0040260.1	*BC11B*	Ola0061540.1	*LGUL*
Ola0196300.1	*ACINU*	Ola0041900.1	*BAZ1B*	Ola0078650.1	*LGR4*
Ola0025860.1	*ACHG*	Ola0152420.1	*BAT1*	Ola0117150.1	*GRINA*
Ola0227960.1	*CKS2*	Ola0226970.1	*BASI*	Ola0187260.1	*LEGL*
Ola0071470.1	*CK5P2*	Ola0096530.1	*B4GN1*	Ola0071000.1	*ARG28*
Ola0072530.1	*CIZ1*	Ola0012980.1	*B3GT2*	Ola0040710.1	*LAYN*
Ola0130380.1	*ACHB2*	Ola0229930.1	*B3GN3*	Ola0027300.1	*LAT4*
Ola0010770.1	*ACHA7*	Ola0194870.1	*B3G5A*	Ola0130520.1	*SLC43A1*
Ola0071340.1	*CI114*	Ola0100130.1	*B2L14*	Ola0061230.1	*LARP6*
Ola0072540.1	*CI016*	Ola0071030.1	*RRF2M*	Ola0071180.1	*LAMC3*
Ola0068620.1	*CHSS3*	Ola0211240.1	*RRBP1*	Ola0069580.1	*LAGE3*
Ola0079760.1	*CHS1*	Ola0070390.1	*ATS6*	Ola0086780.1	*L2GL2*
Ola0196250.1	*CHD8*	Ola0071400.1	*RPGF1*	Ola0104810.1	*L1CAM*
Ola0040980.1	*CHCH2*	Ola0229250.1	*RPF1*	Ola0079800.1	*KS6A2*
Ola0127790.1	*CFA43*	Ola0028600.1	*CRCP*	Ola0040940.1	*KMT2A*
Ola0069650.1	*CETN3*	Ola0086670.1	*RPC4*	Ola0027930.1	*KLOT*
Ola0228710.1	*CERS1*	Ola0078870.1	*RPAB5*	Ola0071930.1	*KLHL8*
Ola0078230.1	*CENPQ*	Ola0181770.1	*RP3A*	Ola0099690.1	*KLD7A*
Ola0099100.1	*CELR2*	Ola0134000.1	*ROAA*	Ola0193380.1	*KIF2C*
Ola0227090.1	*CELF5*	Ola0195060.1	*RO60*	Ola0070310.1	*KIF2A*
Ola0082770.1	*CEGT*	Ola0079790.1	*RNT2*	Ola0079300.1	*KI26B*
Ola0068450.1	*CE030*	Ola0130020.1	*RNH2C*	Ola0078630.1	*KI18A*
Ola0068930.1	*CDK9*	Ola0130390.1	*RNF38*	Ola0061510.1	*KI13B*
Ola0105920.1	*CDK17*	Ola0040920.1	*RNF26*	Ola0069740.1	*ARBK2*
Ola0195050.1	*CDC73*	Ola0077720.1	*RN219*	Ola0229840.1	*KDM4B*
Ola0188220.1	*CDC5L*	Ola0025800.1	*RN167*	Ola0133950.1	*KDM3B*
Ola0000340.1	*CDC26*	Ola0071620.1	*RN165*	Ola0130430.1	*KDM2A*
Ola0068720.1	*CD63*	Ola0117000.1	*RN146*	Ola0136340.1	*KCTD6*
Ola0226420.1	*ZYG11*	Ola0226960.1	*RN126*	Ola0071510.1	*KCNT1*
Ola0070320.1	*ZSWM6*	Ola0025730.1	*RN121*	Ola0229860.1	*KCNN2*
Ola0040720.1	*ZPLD1*	Ola0229090.1	*RM47*	Ola0040420.1	*KCNKD*

Ola0099450.1	*ZP3R*	Ola0065080.1	*RM42*	Ola0118320.1	*KCNK5*
Ola0078810.1	*ZO1*	Ola0040370.1	*RM28*	Ola0129960.1	*KCNK2*
Ola0082310.1	*SLC30A5*	Ola0134490.1	*RM18*	Ola0105880.1	*KCND3*
Ola0040380.1	*ZNT1*	Ola0078220.1	*RM16*	Ola0104430.1	*KCNC4*
Ola0117990.1	*ZN665*	Ola0100120.1	*RLA2*	Ola0099340.1	*KCNB1*
Ola0072470.1	*ZN618*	Ola0071300.1	*RL7A*	Ola0067930.1	*KCNA5*
Ola0118080.1	*ZN555*	Ola0227110.1	*RL3R1*	Ola0010720.1	*KCNA4*
Ola0072040.1	*ZN532*	Ola0071590.1	*RL37*	Ola0100160.1	*KCNA3*
Ola0118060.1	*ZN227*	Ola0187030.1	*AATC*	Ola0100150.1	*KCNA2*
Ola0118070.1	*ZN208*	Ola0071560.1	*RL28*	Ola0071730.1	*KCMF1*
Ola0196220.1	*ZN157*	Ola0068030.1	*RL18A*	Ola0067910.1	*KCMB4*
Ola0152390.1	*ZMYM4*	Ola0025970.1	*GRK1*	Ola0194910.1	*KCMB3*
Ola0025620.1	*CCHCR*	Ola0025710.1	*RIR1*	Ola0194890.1	*KCMB2*
Ola0059930.1	*ZFHX3*	Ola0116150.1	*RIMS2*	Ola0186910.1	*KCIP2*
Ola0060250.1	*ZFAN3*	Ola0182020.1	*AAK1*	Ola0100140.1	*KCA10*
Ola0060210.1	*ZDHC7*	Ola0104820.1	*RIBC1*	Ola0026180.1	*KAT5*
Ola0186710.1	*ZDHC6*	Ola0079570.1	*RHOU*	Ola0071330.1	*KAT3*
Ola0130560.1	*ZDHHC5*	Ola0040500.1	*RHG35*	Ola0229290.1	*KAPCB*
Ola0069100.1	*ZDH12*	Ola0071960.1	*RHG24*	Ola0070880.1	*KANK1*
Ola0069610.1	*ZCHC9*	Ola0186720.1	*RHG22*	Ola0193470.1	*K1614*
Ola0130400.1	*ZCHC7*	Ola0072480.1	*RGS3*	Ola0079920.1	*K154L*
Ola0134520.1	*ZBTB5*	Ola0195080.1	*RGS21*	Ola0067130.1	*K132L*
Ola0130030.1	*ZBTB3*	Ola0195090.1	*RGS18*	Ola0232250.1	*K1257*
Ola0072160.1	*ZBT7C*	Ola0195070.1	*RGS13*	Ola0068710.1	*K102L*
Ola0195030.1	*ZBT41*	Ola0226390.1	*RGPS2*	Ola0068020.1	*K0930*
Ola0074020.1	*ZBT26*	Ola0068780.1	*RGPS1*	Ola0226340.1	*JUN*
Ola0187960.1	*ZBT18*	Ola0133570.1	*RF12A*	Ola0074010.1	*JAK2*
Ola0027950.1	*ZAR1*	Ola0227350.1	*REX1B*	Ola0040240.1	*JAG1B*
Ola0028460.1	*CCD92*	Ola0040360.1	*RELB*	Ola0079360.1	*ITSN2*
Ola0104900.1	*Z385A*	Ola0195180.1	*REL3*	Ola0098700.1	*ITIH3*
Ola0193290.1	*Z354A*	Ola0133940.1	*REEP2*	Ola0071740.1	*ITA1*
Ola0136350.1	*CCD71*	Ola0099330.1	*RDH10*	Ola0005410.1	*ISPD*
Ola0086640.1	*CCD57*	Ola0079840.1	*RCN1*	Ola0060380.1	*ISL3*
Ola0000310.1	*YTDC1*	Ola0074030.1	*RC3H2*	Ola0133560.1	*ACOD1*
Ola0069090.1	*CCD42*	Ola0193480.1	*RC3H1*	Ola0099270.1	*IRF6*
Ola0071990.1	*CCBE1*	Ola0025690.1	*RB1*	Ola0060170.1	*IRF2*
Ola0193490.1	*YOF5*	Ola0079020.1	*RBTN1*	Ola0107720.1	*IQEC2*
Ola0129780.1	*YIF1A*	Ola0188180.1	*RBM45*	Ola0232230.1	*IQEC1*
Ola0068190.1	*CC136*	Ola0077730.1	*RBM26*	Ola0195210.1	*IPP*
Ola0229870.1	*CC124*	Ola0104400.1	*RBM15*	Ola0086690.1	*IPMK*
Ola0106700.1	*YI036*	Ola0211250.1	*RASM*	Ola0040210.1	*IP3KC*
Ola0105300.1	*CBX5*	Ola0181380.1	*RASL1*	Ola0130010.1	*IP3KB*
Ola0187520.1	*YCP6*	Ola0130450.1	*RAN*	Ola0118330.1	*EPB41*
Ola0196260.1	*CBLN1*	Ola0025310.1	*RADX*	Ola0065050.1	*INT13*

Ola0086720.1	*CAVN1*	Ola0130470.1	*RAD9A*	Ola0027280.1	*INP5K*
Ola0136360.1	*DDB*	Ola0072110.1	*RAD1*	Ola0040630.1	*IFNAR1*
Ola0103820.1	*CASZ1*	Ola0072490.1	*RABEK*	Ola0079810.1	*IMP1L*
Ola0196210.1	*CASQ1*	Ola0040480.1	*RAB4B*	Ola0068490.1	*IL6RB*
Ola0040130.1	*CASP*	Ola0028210.1	*RAB34*	Ola0070770.1	*IL31R*
Ola0098730.1	*CAR19*	Ola0060220.1	*RGS9BP*	Ola0025740.1	*IL1AP*
Ola0226380.1	*XYLK*	Ola0130580.1	*R4RL2*	Ola0099700.1	*IGS21*
Ola0069620.1	*XRCC4*	Ola0106660.1	*QRIC1*	Ola0039860.1	*IGSF10*
Ola0067990.1	*XPOT*	Ola0040320.1	*QPCT*	Ola0061400.1	*IGF1R*
Ola0099320.1	*XPO2*	Ola0187070.1	*PYROXD2*	Ola0231980.1	*EIF6*
Ola0079380.1	*XKR5*	Ola0026850.1	*PYGM*	Ola0068870.1	*IER5L*
Ola0118040.1	*XFIN*	Ola0065070.1	*PWP1*	Ola0104760.1	*IDH3G*
Ola0079850.1	*WT1A*	Ola0104850.1	*PUT2*	Ola0116370.1	*IBTK*
Ola0077790.1	*WDSUB1*	Ola0068480.1	*PURG*	Ola0082830.1	*APC*
Ola0234100.1	*WNT4A*	Ola0136230.1	*PURA*	Ola0098690.1	*IL17RC*
Ola0104780.1	*WNK3*	Ola0187970.1	*PURA2*	Ola0226550.1	*IL12RB2*
Ola0098710.1	*WNK2*	Ola0229830.1	*PTPRS*	Ola0180820.1	*ANAPC5*
Ola0098240.1	*CADPS*	Ola0068130.1	*PTPRO*	Ola0105250.1	*HOXC8A*
Ola0078910.1	*CAPRIN1*	Ola0098230.1	*PTPRG*	Ola0105260.1	*HOXC6A*
Ola0069590.1	*WDR45*	Ola0106650.1	*PTPRB*	Ola0105270.1	*HOXC5A*
Ola0072990.1	*WDR70*	Ola0068860.1	*PTPA*	Ola0105280.1	*HOXC4*
Ola0040180.1	*WDR53*	Ola0211270.1	*PTPN4*	Ola0104800.1	*HUWE1*
Ola0106690.1	*WDR46*	Ola0166710.1	*PTPN14*	Ola0067140.1	*AMDHD1*
Ola0070570.1	*WDR41*	Ola0071940.1	*PTPN13*	Ola0078830.1	*APBA2*
Ola0082760.1	*WDR36*	Ola0068040.1	*PTHLH*	Ola0133520.1	*HTXA*
Ola0069130.1	*WDR34*	Ola0099350.1	*PTGIS*	Ola0082800.1	*HSDL2*
Ola0186700.1	*WDFY4*	Ola0071280.1	*PTGES*	Ola0229140.1	*PLAAT1*
Ola0028550.1	*VTN*	Ola0071240.1	*PTCD2*	Ola0025000.1	*HRH2*
Ola0186690.1	*VSTM4*	Ola0193370.1	*PTCH1*	Ola0187010.1	*HPSE2*
Ola0134480.1	*ATP6V0A2*	Ola0071500.1	*PSMD5*	Ola0061410.1	*AP3S2*
Ola0086730.1	*ATP6V0A1*	Ola0136220.1	*PSD2*	Ola0136060.1	*HPRT1*
Ola0071260.1	*NOS1AP*	Ola0039850.1	*AADAC*	Ola0070600.1	*AP3B1*
Ola0181760.1	*VPS33A*	Ola0129790.1	*ATLA3*	Ola0069640.1	*HAPLN1*
Ola0095300.1	*VPS13B*	Ola0068830.1	*PSMB7*	Ola0040490.1	*AP2S1*
Ola0229940.1	*VLDLR*	Ola0100100.1	*ADORA3*	Ola0105290.1	*HOX3*
Ola0073650.1	*GGCX*	Ola0071690.1	*PRUNE2*	Ola0070350.1	*HNRNPD*
Ola0229210.1	*VIT2*	Ola0116320.1	*PRSS35*	Ola0070460.1	*HMGCR*
Ola0229200.1	*VTG1*	Ola0129810.1	*PRSS23*	Ola0116330.1	*SNAP91*
Ola0196170.1	*VIPR1*	Ola0068650.1	*PRRC1*	Ola0061520.1	*HMBOX1*
Ola0116290.1	*VIM*	Ola0000350.1	*PRPF4*	Ola0059380.1	*URAH*
Ola0181410.1	*VGLL4*	Ola0134470.1	*PRPF19*	Ola0082300.1	*ANXA1*
Ola0067190.1	*VEZT*	Ola0059940.1	*PRP16*	Ola0070790.1	*ANKRA2*
Ola0232910.1	*CAMKV*	Ola0194990.1	*PFN2*	Ola0227980.1	*ANKRD24*
Ola0040430.1	*CALM2B*	Ola0100110.1	*ADORA1*	Ola0229190.1	*NPR2*

Ola0071520.1	*CAMSAP1B*	Ola0129990.1	*ATG2A*	Ola0065060.1	*ANO6*
Ola0228480.1	*CALR*	Ola0072070.1	*PRLR*	Ola0079910.1	*HIPK3*
Ola0082750.1	*ATP6V1G1*	Ola0040590.1	*PRLHR*	Ola0041880.1	*HIP1*
Ola0060040.1	*VAT1L*	Ola0106630.1	*PRICKLE2*	Ola0026160.1	*HINFP*
Ola0105800.1	*ATP6AP1*	Ola0226330.1	*PRDX6*	Ola0071020.1	*HEXB*
Ola0104750.1	*AVPR2*	Ola0116300.1	*PRDM13*	Ola0103940.1	*HES4B*
Ola0079220.1	*USH2A*	Ola0070410.1	*PPWD1*	Ola0116810.1	*HPN*
Ola0229270.1	*UOX*	Ola0040390.1	*PPPLR37*	Ola0092820.1	*ANKMY2*
Ola0231960.1	*UQCC1*	Ola0069190.1	*PPP6C*	Ola0181780.1	*HECTD4*
Ola0028580.1	*CA4*	Ola0038770.1	*PPP5C*	Ola0040770.1	*ANKK1*
Ola0070950.1	*UNC5D*	Ola0193430.1	*PPM1L*	Ola0226980.1	*HCN2*
Ola0227130.1	*UNC13A*	Ola0079580.1	*PPM1G*	Ola0071980.1	*HCN1*
Ola0116920.1	*ULK4*	Ola0071760.1	*PSTPIP2*	Ola0226400.1	*ANGPTL1*
Ola0061260.1	*ULK3*	Ola0060390.1	*PSTPIP1*	Ola0086700.1	*NAGLU*
Ola0194970.1	*FYTTD1*	Ola0069140.1	*PPIA*	Ola0026130.1	*H2AFX*
Ola0229850.1	*UHRF1*	Ola0106640.1	*PPHLN1*	Ola0130420.1	*ANKRD13D*
Ola0187230.1	*UGP2*	Ola0099290.1	*PPE34*	Ola0025920.1	*GUCY2F*
Ola0000320.1	*UFC1*	Ola0071900.1	*PPEF1*	Ola0099280.1	*GUCA1A*
Ola0071410.1	*UCK1*	Ola0118310.1	*PON2*	Ola0010730.1	*CGBA*
Ola0099150.1	*USP49*	Ola0012960.1	*Gag-POL*	Ola0072030.1	*GRP*
Ola0060060.1	*USP47*	Ola0069830.1	*PNX*	Ola0069810.1	*GRK5*
Ola0067170.1	*USP44*	Ola0227220.1	*ATP8B1*	Ola0040890.1	*GRIK4*
Ola0071290.1	*USP20*	Ola0061500.1	*PNOC*	Ola0025610.1	*GRIA3*
Ola0078480.1	*CDH15*	Ola0133990.1	*PHYKPL*	Ola0134510.1	*GRHPR*
Ola0068850.1	*CRAT*	Ola0130540.1	*PMF1*	Ola0061420.1	*ANPEP*
Ola0104840.1	*CACNA1S*	Ola0025840.1	*ATP1B2*	Ola0117220.1	*GRB10*
Ola0228520.1	*CACNA1E*	Ola0073990.1	*PLGRKT*	Ola0026150.1	*DPAGT1*
Ola0098760.1	*CACNA1D*	Ola0226320.1	*PLPP6*	Ola0099250.1	*AMPD2*
Ola0028180.1	*CALN1*	Ola0228020.1	*PLPP2*	Ola0099240.1	*GPR61*
Ola0180770.1	*CABP1*	Ola0071790.1	*PLPP1*	Ola0040580.1	*GPR4*
Ola0040950.1	*UBE4A*	Ola0116550.1	*PLEC*	Ola0059960.1	*GPR21*
Ola0116350.1	*UBE3D*	Ola0025870.1	*PLD2*	Ola0193540.1	*GPC5*
Ola0065090.1	*UBE2N*	Ola0188240.1	*PLCB1*	Ola0136090.1	*GPC3*
Ola0136080.1	*CAB39*	Ola0071070.1	*AGPAT1*	Ola0077760.1	*GPALPP1*
Ola0226600.1	*UAP1*	Ola0065110.1	*PKP2*	Ola0071750.1	*GOLPH3*
Ola0069080.1	*UAP1L1*	Ola0069110.1	*PKN2*	Ola0072510.1	*GOLGA2*
Ola0105810.1	*CACNA2D3*	Ola0068440.1	*PLEKHA7*	Ola0078190.1	*BLZF1*
Ola0027260.1	*SLC25A1*	Ola0103910.1	*PIK3CD*	Ola0061550.1	*GNRHR2*
Ola0078210.1	*TXNL4B*	Ola0194900.1	*PIK3CA*	Ola0060580.1	*GNB5B*
Ola0103830.1	*CA127*	Ola0186940.1	*PK1R*	Ola0073770.1	*GNAZ*
Ola0028500.1	*TUSC5*	Ola0070300.1	*PITPNM2*	Ola0071700.1	*GNAQ*
Ola0229300.1	*TTLL7*	Ola0027290.1	*PITPNA*	Ola0073660.1	*GMCL1*
Ola0129770.1	*CCDC85CA*	Ola0179110.1	*PIM2*	Ola0099230.1	*AMIGO1*
Ola0187080.1	*TTC31*	Ola0027160.1	*PIGS*	Ola0026670.1	*AMER2*

Ola0040760.1	*TTC12*	Ola0227200.1	*PIAS4*	Ola0078900.1	*ALX4*
Ola0116280.1	*TSTD3*	Ola0073590.1	*INPP5J*	Ola0104410.1	*ALX3*
Ola0071660.1	*THBS4B*	Ola0040440.1	*PTGIR*	Ola0180790.1	*GGT5*
Ola0133540.1	*ML1136*	Ola0071440.1	*PHYHD1*	Ola0180810.1	*GGT1*
Ola0118100.1	*TRY3*	Ola0194960.1	*PHR*	Ola0182060.1	*GFPT1*
Ola0098320.1	*TRUB2*	Ola0071570.1	*PDCL*	Ola0040410.1	*GEMIN7*
Ola0130040.1	*TRPT1*	Ola0026680.1	*PHLDB2*	Ola0116430.1	*GDF6A*
Ola0060560.1	*TRPM7*	Ola0026120.1	*PHLDB1*	Ola0231950.1	*GDF5*
Ola0070470.1	*COL4A3BP*	Ola0040990.1	*PHKG1*	Ola0068140.1	*AT3G55350*
Ola0060180.1	*CDC42SE2*	Ola0104790.1	*PHF8*	Ola0072050.1	*ALPK2*
Ola0025700.1	*TRPC2*	Ola0136070.1	*PHF6*	Ola0166640.1	*ACBD3*
Ola0026140.1	*C2CD2L*	Ola0071580.1	*PTGS1*	Ola0070450.1	*GCNT4*
Ola0077740.1	*CYP27C1*	Ola0067970.1	*PHF21B*	Ola0079350.1	*GCFC2*
Ola0070530.1	*CH25HL2*	Ola0222440.1	*PESL*	Ola0026690.1	*GABRR3*
Ola0040930.1	*C1QTNF5*	Ola0073620.1	*EPX*	Ola0024860.1	*GABRB2*
Ola0104860.1	*C1QL4*	Ola0061200.1	*PEPD*	Ola0024850.1	*GABRA3*
Ola0096510.1	*C1GALT1B*	Ola0187240.1	*PELI1*	Ola0134020.1	*GNB2L1*
Ola0116970.1	*TRMT11*	Ola0071220.1	*PTGER4*	Ola0229240.1	*GNG5*
Ola0079030.1	*TRIM66*	Ola0127820.1	*PDZD7*	Ola0229120.1	*GNB4*
Ola0196230.1	*TRIM25*	Ola0073470.1	*ZNF33A*	Ola0025890.1	*GNB2*
Ola0070420.1	*TRIM23*	Ola0072570.1	*ASS1*	Ola0061270.1	*GAS1*
Ola0099200.1	*TRIM16*	Ola0027940.1	*PDS5B*	Ola0071270.1	*GARNL3*
Ola0196270.1	*TRIM11*	Ola0070560.1	*PDE8B*	Ola0222430.1	*GAL3ST1*
Ola0065040.1	*TRHR*	Ola0059370.1	*PDCD5*	Ola0041910.1	*FZD9*
Ola0116910.1	*TRAK1*	Ola0078240.1	*PTGDR2*	Ola0119240.1	*ALG2*
Ola0129980.1	*TRMT112*	Ola0073980.1	*CD274*	Ola0040150.1	*FUT9*
Ola0028610.1	*TPST1*	Ola0025040.1	*PCDH1*	Ola0226950.1	*FSTL3*
Ola0068500.1	*TPM1*	Ola0025010.1	*PCDHAC2*	Ola0027960.1	*FRY*
Ola0099130.1	*BYSL*	Ola0025030.1	*PCDH10*	Ola0133500.1	*FRMPD3*
Ola0070970.1	*TRAPPC13*	Ola0099310.1	*PBRM1*	Ola0186740.1	*FRMPD2*
Ola0116360.1	*TPBG*	Ola0180780.1	*ASPHD2*	Ola0134530.1	*FRMPD1*
Ola0196240.1	*TOX4*	Ola0079830.1	*PAX6*	Ola0222480.1	*FOXN4*
Ola0071050.1	*TOR1B*	Ola0166670.1	*PARP1*	Ola0103930.1	*FOXL2*
Ola0070960.1	*TNFRSF26*	Ola0193510.1	*PARL*	Ola0070810.1	*FOXD2*
Ola0070830.1	*TNPO1*	Ola0070490.1	*F2RL2*	Ola0227210.1	*FOXA3*
Ola0025880.1	*TNIK*	Ola0070520.1	*F2RL1*	Ola0129760.1	*FOSL2*
Ola0226370.1	*FASLG*	Ola0070500.1	*PAR1*	Ola0040270.1	*FOSB*
Ola0130550.1	*TMX2B*	Ola0104970.1	*ANKRD33*	Ola0027180.1	*ALDOCB*
Ola0040620.1	*MPRSS13*	Ola0187530.1	*PANK1*	Ola0071080.1	*FNBP1*
Ola0086760.1	*TMEM94*	Ola0226570.1	*SERBP1*	Ola0226290.1	*FMO5*
Ola0116460.1	*TMEM74*	Ola0071550.1	*PAB1*	Ola0071250.1	*CACFD1*
Ola0227330.1	*TMEM59*	Ola0078450.1	*PABPN1*	Ola0229110.1	*ALDH7A1*
Ola0027210.1	*TMIGD1*	Ola0070750.1	*PIK3R1*	Ola0028570.1	*ALDH3A2*
Ola0067220.1	*TMCC3*	Ola0195200.1	*PIK3R3*	Ola0010740.1	*ARL14EP*

Ola0099160.1	*TMCC2*	Ola0039870.1	*P2RY12*	Ola0227310.1	*FKBP8*
Ola0082290.1	*TMC2A*	Ola0130490.1	*P2RX3*	Ola0098300.1	*FKBP5*
Ola0061240.1	*TM2D3*	Ola0222450.1	*P2RX2*	Ola0129750.1	*FIBPB*
Ola0103920.1	*TMEM201*	Ola0069820.1	*ASH2L*	Ola0195040.1	*CFHR1*
Ola0040100.1	*BUD23*	Ola0070580.1	*OTPB*	Ola0061460.1	*AKAP13*
Ola0211260.1	*TMEM177*	Ola0026660.1	*SLC51A*	Ola0193520.1	*FGF12*
Ola0040510.1	*TMEM160*	Ola0228490.1	*OSBPL9*	Ola0067200.1	*FGD6*
Ola0078200.1	*TRMT10C*	Ola0096520.1	*OS9*	Ola0195000.1	*AHSG*
Ola0182040.1	*TLS1*	Ola0025930.1	*SLC25A15*	Ola0000360.1	*FER*
Ola0228040.1	*TLE1*	Ola0025770.1	*OR6C6*	Ola0181430.1	*AJM1*
Ola0098750.1	*TKT*	Ola0105910.1	*OPSP*	Ola0070820.1	*FCHO2*
Ola0104910.1	*TAC3*	Ola0098270.1	*OPSG*	Ola0073500.1	*FCGBP*
Ola0133910.1	*TIMD4*	Ola0106680.1	*OGG1*	Ola0068790.1	*FBXW2*
Ola0025640.1	*TIGD1*	Ola0116390.1	*BCKDHB*	Ola0068010.1	*FBXO7*
Ola0099360.1	*THUMPD3*	Ola0227950.1	*ODF3L2*	Ola0028470.1	*FBXO40*
Ola0061210.1	*THSD4*	Ola0204560.1	*OCLN*	Ola0071170.1	*FIBCD1*
Ola0118120.1	*BTG3*	Ola0025790.1	*OR51E1*	Ola0086630.1	*FASN*
Ola0070800.1	*BTF3*	Ola0193530.1	*NYAP2*	Ola0104770.1	*FAM3A*
Ola0069710.1	*ACADS*	Ola0025830.1	*NYAP1*	Ola0028480.1	*AIFM3*
Ola0072410.1	*THAP5*	Ola0130000.1	*NXF1*	Ola0040970.1	*FAM98C*
Ola0082330.1	*THAP1*	Ola0070630.1	*ARSB*	Ola0118350.1	*AIF1L*
Ola0232240.1	*ACAD9*	Ola0069670.1	*ARRDC3*	Ola0227360.1	*FAM78B*
Ola0129970.1	*TGFB2*	Ola0071890.1	*NUP54*	Ola0025670.1	*FAM76B*
Ola0038760.1	*TGFB1*	Ola0067980.1	*NUP50*	Ola0133980.1	*FAM53C*
Ola0079330.1	*TFB2M*	Ola0060600.1	*ARPP19*	Ola0025340.1	*FAM46C*
Ola0086680.1	*TFAM*	Ola0060230.1	*NUD19*	Ola0116380.1	*FAM46A*
Ola0222470.1	*ACACB*	Ola0065100.1	*NUDT11*	Ola0073000.1	*FAM222A*
Ola0072100.1	*BRIX1*	Ola0099120.1	*NUAK2*	Ola0060610.1	*FAM214A*
Ola0060200.1	*TERF2*	Ola0071460.1	*NUP188*	Ola0070890.1	*FBP1*
Ola0193300.1	*TENR*	Ola0071720.1	*NTRK2*	Ola0204590.1	*FAM133*
Ola0025330.1	*BRWD3*	Ola0071390.1	*NTNG2*	Ola0136050.1	*FAM122A*
Ola0060090.1	*TEAD1*	Ola0068880.1	*NTMT1*	Ola0079610.1	*FAM120B*
Ola0086750.1	*TDRKH*	Ola0028220.1	*NSRP1*	Ola0069060.1	*FAM102A*
Ola0018600.1	*BRINP3*	Ola0029010.1	*NSG1*	Ola0193500.1	*EXTL2*
Ola0027190.1	*CCT8*	Ola0071040.1	*NSA2*	Ola0078890.1	*EXT2*
Ola0072420.1	*BRINP1*	Ola0026860.1	*NRXN2*	Ola0079370.1	*EVA1A*
Ola0059330.1	*TCF25*	Ola0117380.1	*NRSN1*	Ola0072080.1	*AGXT2*
Ola0195190.1	*DYNLT5*	Ola0136240.1	*NRG2*	Ola0099260.1	*EPS8L3*
Ola0072770.1	*TBX5A*	Ola0068460.1	*NRG1*	Ola0133530.1	*ES1*
Ola0072760.1	*TBX3*	Ola0152370.1	*NRDE2*	Ola0079210.1	*ERR3*
Ola0194880.1	*TBL1XR1*	Ola0068840.1	*NR5A2*	Ola0119270.1	*ERP44*
Ola0040340.1	*TBCB*	Ola0119250.1	*NR4A3*	Ola0026700.1	*ADGRG2*
Ola0070590.1	*TBCA*	Ola0204570.1	*NR2F6*	Ola0193400.1	*ERICH6*
Ola0152380.1	*TBB*	Ola0129740.1	*NPAS4A*	Ola0077750.1	*ERCC3*

Ola0077780.1	*TANC1*	Ola0187510.1	*NPHP1*	Ola0098680.1	*ERC2*
Ola0193460.1	*TAL1*	Ola0070870.1	*NPFFR2*	Ola0070990.1	*ERBIN*
Ola0026000.1	*TAF6*	Ola0040520.1	*NPAS3*	Ola0068120.1	*EPS8*
Ola0025940.1	*TM4SF1*	Ola0025320.1	*NPAS2*	Ola0194930.1	*EPHB3*
Ola0077770.1	*GTF2F2*	Ola0227380.1	*NOTCH2*	Ola0116440.1	*ENY2B*
Ola0194980.1	*TSC22D2*	Ola0071530.1	*NOTCH1A*	Ola0086660.1	*ENPP7*
Ola0226840.1	*TMEM161A*	Ola0194950.1	*NMUR1*	Ola0071010.1	*ENC1*
Ola0061250.1	*TARS3*	Ola0025750.1	*NLRC3*	Ola0116470.1	*EMC2*
Ola0078840.1	*SYT7*	Ola0025630.1	*NLGN3*	Ola0079280.1	*AHCTF1*
Ola0106670.1	*QARS*	Ola0027270.1	*NLE1*	Ola0079820.1	*ELP4*
Ola0098250.1	*SYNPR*	Ola0187050.1	*NKX32*	Ola0227300.1	*ELL*
Ola0181420.1	*SYN2*	Ola0187040.1	*NKX23*	Ola0228400.1	*ELAVL4*
Ola0071910.1	*BMP4*	Ola0061010.1	*NKD1*	Ola0116480.1	*EIF3EA*
Ola0040400.1	*BMP2*	Ola0069790.1	*TACR1*	Ola0196190.1	*EIF2B5*
Ola0073460.1	*BMP1*	Ola0041010.1	*NIPSNAP2*	Ola0222390.1	*EHD2*
Ola0061530.1	*EPRS*	Ola0060190.1	*NIP7*	Ola0133930.1	*EGR1*
Ola0070340.1	*SYB*	Ola0098720.1	*NINJ1*	Ola0040530.1	*EGLN2*
Ola0082320.1	*SOWAHB*	Ola0166630.1	*NHSL1*	Ola0071060.1	*EGFL7*
Ola0234110.1	*SVBP*	Ola0100170.1	*NGF*	Ola0166690.1	*EFR3B*
Ola0070480.1	*SV2C*	Ola0182050.1	*NFU1*	Ola0228050.1	*AES*
Ola0061450.1	*SV2B*	Ola0227230.1	*NFIL3*	Ola0078250.1	*EFNB2A*
Ola0071320.1	*SURF6*	Ola0133580.1	*NEXMIF*	Ola0069840.1	*AEBP1*
Ola0072560.1	*SURF4*	Ola0040190.1	*NEU4*	Ola0040200.1	*GFM1*
Ola0072550.1	*SURF2*	Ola0067160.1	*NTN4*	Ola0227120.1	*EEF2*
Ola0071310.1	*SURF1*	Ola0068820.1	*NEK6*	Ola0226430.1	*ECHDC2*
Ola0228500.1	*ABL2*	Ola0072060.1	*NEDD4L*	Ola0117010.1	*ECHDC1*
Ola0039840.1	*SUCNR1*	Ola0229080.1	*NDUFB5*	Ola0072520.1	*DNM1*
Ola0130440.1	*STX3*	Ola0067210.1	*NDUFA12*	Ola0072130.1	*DYM*
Ola0040090.1	*STX1A*	Ola0229950.1	*NDUFA7*	Ola0012950.1	*DNAH6*
Ola0119260.1	*STX17*	Ola0068890.1	*NDOR1*	Ola0099110.1	*DSTYK*
Ola0099190.1	*STRIP1*	Ola0134010.1	*NME5*	Ola0181390.1	*DTX1*
Ola0130410.1	*STAP1*	Ola0228720.1	*NCLN*	Ola0040610.1	*DSCAML1*
Ola0086710.1	*STAT5B*	Ola0105490.1	*NAV1*	Ola0133920.1	*DSCAM*
Ola0130500.1	*SSRP1*	Ola0069070.1	*NAIF1*	Ola0186680.1	*DRGX*
Ola0134500.1	*SSH*	Ola0070430.1	*MZT2*	Ola0105890.1	*DRD2*
Ola0227320.1	*SSBP3*	Ola0118000.1	*MYPOP*	Ola0040780.1	*D215*
Ola0099170.1	*SRSF3*	Ola0078660.1	*MYBPC3*	Ola0029080.1	*DRD1L*
Ola0025810.1	*SRRT*	Ola0228750.1	*MYO9B*	Ola0181750.1	*DPYSL2*
Ola0040170.1	*SRPRB*	Ola0227140.1	*MYO5A*	Ola0222460.1	*DPOE*
Ola0099370.1	*SRGAP3*	Ola0222490.1	*MYO1H*	Ola0227290.1	*DOT1L*
Ola0070780.1	*SREK1*	Ola0086770.1	*MYO15*	Ola0071380.1	*DOLPP1*
Ola0069150.1	*SPTAN1*	Ola0104870.1	*MYL6*	Ola0116340.1	*DOPEY1*
Ola0028200.1	*SUPT6H*	Ola0069750.1	*MYO18B*	Ola0071450.1	*DOLK*
Ola0026710.1	*SMS*	Ola0059360.1	*MVD*	Ola0107740.1	*DNASE1L2*

Ola0228410.1	*SPRR1A*	Ola0079290.1	*MUM1*	Ola0229260.1	*DNASE2B*
Ola0073760.1	*SPPL3*	Ola0196180.1	*MUL1A*	Ola0071420.1	*DNLZ*
Ola0133960.1	*SPON2*	Ola0040910.1	*MCAM*	Ola0071650.1	*DNAJB5*
Ola0079930.1	*SPON1*	Ola0071780.1	*MTREX*	Ola0228420.1	*DMRTA2*
Ola0078670.1	*SPI1*	Ola0025660.1	*MTMR2*	Ola0072210.1	*ADD1*
Ola0229150.1	*FTSJ3*	Ola0078460.1	*CBFA2T3*	Ola0079600.1	*DLD*
Ola0229230.1	*SPATA1*	Ola0136040.1	*MOSPD1*	Ola0060070.1	*DKK3*
Ola0104950.1	*SP5*	Ola0059340.1	*MC1R*		

Table S4. Candidate genes positively selected in overlap in both *O. lacepedii* and *O. rebecca* ($p < 0.05$).

Gene ID	Swissprot Annotation	Gene ID	Swissprot Annotation	Gene ID	Swissprot Annotation
Ola0018600.1	*BRINP3*	Ola0105250.1	*HOXC8A*	Ola0214070.1	*SIK2*
Ola0025610.1	*GRIA3*	Ola0105260.1	*HOXC6A*	Ola0214650.1	*PHF6*
Ola0026120.1	*PHLDB1*	Ola0105650.1	*KCNA3*	Ola0214980.1	*ES1*
Ola0104430.1	*KCNC4*	Ola0116280.1	*TSTD3*	Ola0214990.1	*ACOD1*
Ola0028600.1	*CRCP*	Ola0116290.1	*VIM*	Ola0221250.1	*KCNIP3*
Ola0028610.1	*TPST1*	Ola0116300.1	*PRDM13*	Ola0221300.1	*ADRA2B*
Ola0028620.1	*OPSP*	Ola0118120.1	*BTG3*	Ola0221370.1	*OTPB*
Ola0038700.1	*RYR1*	Ola0118130.1	*KCNN2*	Ola0221380.1	*SCAMP1*
Ola0040780.1	*D215*	Ola0181420.1	*SYN2*	Ola0222200.1	*DNAJB5*
Ola0053220.1	*SLC43A1*	Ola0182510.1	*CMKLR1*	Ola0223790.1	*RGS9BP*
Ola0053230.1	*ZDHHC5*	Ola0187900.1	*ARID5B*	Ola0227900.1	*FOXD2*
Ola0059700.1	*NEDD4L*	Ola0188180.1	*RBM45*	Ola0227940.1	*CPAMD8*
Ola0061380.1	*SNAP25A*	Ola0188190.1	*CYCS*	Ola0227960.1	*CKS2*
Ola0073350.1	*LRRC75A*	Ola0194070.1	*HCN2*	Ola0227970.1	*SHC2*
Ola0104450.1	*AHCYL2*	Ola0195050.1	*CDC73*	Ola0228040.1	*TLE1*
Ola0082310.1	*SLC30A5*	Ola0195060.1	*RO60*	Ola0228050.1	*AES*
Ola0082320.1	*SOWAHB*	Ola0195070.1	*RGS13*	Ola0228060.1	*GNAQ*
Ola0095300.1	*VPS13B*	Ola0195080.1	*RGS21*	Ola0228220.1	*TPM1*
Ola0104180.1	*GPR61*	Ola0195090.1	*RGS18*	Ola0228410.1	*SPRR1A*
Ola0104190.1	*AMIGO1*	Ola0209440.1	*GRINA*	Ola0228420.1	*DMRTA2*

Table S5. GO enrichment of positively selected genes in *O. lacepedii* ($p < 0.05$).

GO ID	Description	Count	Gene ratio	BgRatio	P value
GO:0042775	mitochondrial ATP synthesis coupled electron transport	5	5/529	21/19107	3.16E-04
GO:0030976	Thiamine pyrophosphate binding	5	5/529	25/19107	6.88E-04
GO:0022008	neurogenesis	3	3/529	7/19107	1.01E-03
GO:0019842	vitamin binding	6	6/529	42/19107	1.30E-03
GO:0006091	generation of precursor metabolites and energy	15	15/529	217/19107	1.80E-03
GO:0016829	lyase activity	19	19/529	312/19107	1.97E-03
GO:0006189	'de novo' IMP biosynthetic process	2	2/529	3/19107	1.99E-03
GO:0015980	energy derivation by oxidation of organic compounds	10	10/529	116/19107	2.08E-03
GO:0016043	cellular component organization	60	60/529	1462/19107	2.20E-03
GO:0022900	electron transport chain	9	9/529	100/19107	2.34E-03
GO:0017108	5'-flap endonuclease activity	2	2/529	3/19107	2.34E-03
GO:0033557	Slx1-Slx4 complex	2	2/529	3/19107	2.34E-03
GO:0048256	flap endonuclease activity	2	2/529	3/19107	2.34E-03
GO:0004185	serine-type carboxypeptidase activity	3	3/529	11/19107	2.87E-03
GO:0006996	organelle organization	35	35/529	751/19107	2.93E-03
GO:0006119	oxidative phosphorylation	5	5/529	35/19107	3.11E-03
GO:0042773	ATP synthesis coupled electron transport	5	5/529	35/19107	3.11E-03
GO:0016050	vesicle organization	5	5/529	37/19107	3.14E-03
GO:0045333	cellular respiration	9	9/529	105/19107	3.36E-03
GO:0008374	O-acyltransferase activity	4	4/529	21/19107	3.53E-03
GO:0071840	cellular component organization or biogenesis	62	62/529	1560/19107	3.90E-03
GO:0006275	regulation of DNA replication	4	4/529	25/19107	4.04E-03
GO:0005773	vacuole	4	4/529	23/19107	4.08E-03
GO:0030153	bacteriocin immunity	3	3/529	12/19107	5.43E-03
GO:0004664	prephenate dehydratase activity	2	2/529	5/19107	6.97E-03
GO:0009094	L-phenylalanine biosynthetic process	2	2/529	5/19107	6.97E-03
GO:1902223	erythrose 4-phosphate/phosphoenolpyruvate family amino acid biosynthetic process	2	2/529	5/19107	6.97E-03
GO:0016073	snRNA metabolic process	2	2/529	5/19107	7.52E-03
GO:0016180	snRNA processing	2	2/529	5/19107	7.52E-03
GO:0034472	snRNA 3'-end processing	2	2/529	5/19107	7.52E-03

GO:0034477	U6 snRNA 3'-end processing	2	2/529	5/19107	7.52E-03
GO:0043628	ncRNA 3'-end processing	2	2/529	5/19107	7.52E-03
GO:0051052	regulation of DNA metabolic process	4	4/529	30/19107	7.77E-03
GO:0022904	respiratory electron transport chain	6	6/529	61/19107	7.99E-03
GO:0006099	tricarboxylic acid cycle	3	3/529	16/19107	8.11E-03
GO:0015643	toxic substance binding	3	3/529	15/19107	8.67E-03
GO:0006120	mitochondrial electron transport, NADH to ubiquinone	3	3/529	14/19107	8.77E-03
GO:0071705	nitrogen compound transport	10	10/529	147/19107	8.84E-03
GO:0007399	nervous system development	3	3/529	15/19107	9.09E-03
GO:0051260	protein homooligomerization	7	7/529	85/19107	9.51E-03
GO:0000323	lytic vacuole	2	2/529	5/19107	9.87E-03
GO:0005764	lysosome	2	2/529	5/19107	9.87E-03
GO:0006558	L-phenylalanine metabolic M- process	2	2/529	6/19107	1.01E-02
GO:1902221	erythrose 4-phosphate/phosphoenolpyruvate family amino acid metabolic process	2	2/529	6/19107	1.01E-02
GO:0004402	histone acetyltransferase activity	4	4/529	28/19107	1.06E-02
GO:0031090	organelle membrane	27	27/529	592/19107	1.22E-02
GO:0005750	mitochondrial respiratory chain complex III	2	2/529	7/19107	1.33E-02
GO:0006122	mitochondrial electron transport, ubiquinol to cytochrome c	2	2/529	7/19107	1.33E-02
GO:0045275	respiratory chain complex III	2	2/529	7/19107	1.33E-02
GO:0043044	ATP-dependent chromatin remodeling	2	2/529	7/19107	1.35E-02
GO:0061024	membrane organization	11	11/529	183/19107	1.40E-02
GO:0042597	periplasmic space	4	4/529	34/19107	1.44E-02
GO:0016286	small conductance calcium-activated potassium channel activity	2	2/529	7/19107	1.51E-02
GO:0044441	cilium part	2	2/529	7/19107	1.76E-02
GO:0031975	envelope	15	15/529	291/19107	1.79E-02
GO:0016730	oxidoreductase activity, acting on iron-sulfur proteins as donors	2	2/529	7/19107	1.79E-02
GO:0009124	nucleoside monophosphate biosynthetic process	3	3/529	20/19107	1.89E-02
GO:0006611	protein export from nucleus	2	2/529	8/19107	1.90E-02
GO:0006998	nuclear envelope organization	2	2/529	8/19107	1.90E-02

GO:0051259	protein oligomerization	7	7/529	98/19107	1.97E-02
GO:0009123	nucleoside monophosphate metabolic process	3	3/529	21/19107	2.06E-02
GO:0016892	endoribonuclease activity, producing 3'-phosphomonoesters	2	2/529	8/19107	2.20E-02
GO:0009060	aerobic respiration	3	3/529	22/19107	2.23E-02
GO:0006188	IMP biosynthetic process	2	2/529	9/19107	2.27E-02
GO:0046040	IMP metabolic process	2	2/529	9/19107	2.27E-02
GO:0006820	anion transport	11	11/529	188/19107	2.34E-02
GO:0006402	mRNA catabolic process	4	4/529	38/19107	2.34E-02
GO:0009725	response to hormone stimulus	8	8/529	117/19107	2.38E-02
GO:0031967	organelle envelope	14	14/529	278/19107	2.46E-02
GO:0015711	organic anion transport	7	7/529	100/19107	2.53E-02
GO:0015849	organic acid transport	7	7/529	100/19107	2.53E-02
GO:0046942	carboxylic acid transport	7	7/529	100/19107	2.53E-02
GO:0009126	purine nucleoside monophosphate metabolic process	2	2/529	10/19107	2.84E-02
GO:0009127	purine nucleoside monophosphate biosynthetic process	2	2/529	10/19107	2.84E-02
GO:0009156	ribonucleoside monophosphate biosynthetic process	2	2/529	10/19107	2.84E-02
GO:0009161	ribonucleoside monophosphate metabolic process	2	2/529	10/19107	2.84E-02
GO:0009167	purine ribonucleoside monophosphate metabolic process	2	2/529	10/19107	2.84E-02
GO:0009168	purine ribonucleoside monophosphate biosynthetic process	2	2/529	10/19107	2.84E-02
GO:0016836	hydro-lyase activity	4	4/529	43/19107	3.04E-02
GO:0006906	vesicle fusion	2	2/529	10/19107	3.04E-02
GO:0048280	vesicle fusion with Golgi apparatus	2	2/529	1019107	3.04E-02
GO:0004417	hydroxyethylthiazole kinase activity	1	1/529	1/19107	3.08E-02
GO:0050215	propanediol dehydratase activity	1	1/529	1/19107	3.09E-02
GO:0006597	spermine biosynthetic process	1	1/529	1/19107	3.10E-02
GO:0008215	spermine metabolic process	1	1/529	1/19107	3.10E-02
GO:0006401	RNA catabolic process	4	4/529	42/19107	3.15E-02
GO:0031966	mitochondrial membrane	12	12/529	231/19107	3.16E-02
GO:0016163	nitrogenase activity	1	1/529	1/19107	3.18E-02
GO:0016732	oxidoreductase activity, acting on	1	1/529	1/19107	3.18E-02

	iron-sulfur proteins as donors, dinitrogen as acceptor				
GO:0004455	ketol-acid reductoisomerase activity	1	1/529	1/19107	3.19E-02
GO:0000228	nuclear chromosome	7	7/529	108/19107	3.23E-02
GO:0019670	anaerobic glutamate catabolic process	1	1/529	1/19107	3.44E-02
GO:0050097	methylaspartate mutase activity	1	1/529	1/19107	3.44E-02
GO:0005739	mitochondrion	16	16/529	350/19107	3.51E-02
GO:0034655	nucleobase-containing compound catabolic process	7	7/529	106/19107	3.55E-02
GO:0016568	chromatin modification	5	5/529	64/19107	3.64E-02
GO:0004470	malic enzyme activity	2	2/529	10/19107	3.65E-02
GO:0004471	malate dehydrogenase (decarboxylating) activity	2	2/529	10/19107	3.65E-02
GO:0016615	malate dehydrogenase activity	2	2/529	10/19107	3.65E-02
GO:0044454	nuclear chromosome part	6	6/529	90/19107	3.85E-02
GO:0005158	insulin receptor binding	3	3/529	24/19107	3.85E-02
GO:0009719	response to endogenous stimulus	8	8/529	129/19107	3.91E-02
GO:0000184	nuclear-transcribed mRNA catabolic process, nonsense-mediated decay	2	2/529	11/19107	4.02E-02
GO:0015204	urea transmembrane transporter activity	1	1/529	2/19107	4.24E-02
GO:0015840	urea transport	1	1/529	2/19107	4.24E-02
GO:0019755	one-carbon compound transport	1	1/529	2/19107	4.24E-02
GO:0042887	amide transmembrane transporter activity	1	1/529	2/19107	4.24E-02
GO:0071918	urea transmembrane transport	1	1/529	2/19107	4.24E-02
GO:0009095	aromatic amino acid family biosynthetic process, prephenate pathway	2	2/529	12/19107	4.41E-02
GO:0031023	microtubule organizing center organization	2	2/529	12/19107	4.42E-02
GO:0016894	endonuclease activity, active with either ribo- or deoxyribonucleic acids and producing 3'-phosphomonoesters	2	2/529	12/19107	4.43E-02
GO:1901681	sulfur compound binding	5	5/529	66/19107	4.47E-02
GO:0006997	nucleus organization	2	2/529	12/19107	4.47E-02
GO:0000723	telomere maintenance	3	3/529	26/19107	4.48E-02
GO:0032200	telomere organization	3	3/529	26/19107	4.48E-02
GO:0060249	anatomical structure homeostasis	3	3/529	26/19107	4.48E-02
GO:0042221	response to chemical stimulus	13	13/529	262/19107	4.52E-02

GO:0003333	amino acid transmembrane transport	4	4/529	46/19107	4.58E-02
GO:0009110	vitamin biosynthetic process	5	5/529	72/19107	4.66E-02
GO:0042364	water-soluble vitamin biosynthetic process	5	5/529	72/19107	4.66E-02
GO:0016044	cellular membrane organization	8	8/529	144/19107	4.67E-02
GO:0005740	mitochondrial envelope	12	12/529	248/19107	4.69E-02
GO:0061025	membrane fusion	6	6/529	93/19107	4.74E-02
GO:0016852	sirohydrochlorin cobaltochelatase activity	1	1/529	2/19107	4.93E-02

Table S6. GO enrichment of positively selected genes in *O. rebecca* ($p < 0.05$).

GO ID	Description	Count	Gene ratio	BgRatio	P value
GO:1901137	carbohydrate derivative biosynthetic process	48	48/1131	414/19107	1.14E-05
GO:0008378	galactosyltransferase activity	11	11/1131	44/19107	4.12E-05
GO:0055076	transition metal ion homeostasis	7	7/1131	21/19107	8.79E-05
GO:0005227	calcium activated cation channel activity	8	8/1131	28/19107	2.19E-04
GO:0015269	calcium-activated potassium channel activity	8	8/1131	28/19107	2.19E-04
GO:0006879	cellular iron ion homeostasis	6	6/1131	18/19107	2.58E-04
GO:0055072	iron ion homeostasis	6	6/1131	18/19107	2.58E-04
GO:0046916	cellular transition metal ion homeostasis	6	6/1131	19/19107	3.83E-04
GO:0051082	unfolded protein binding	17	17/1131	130/19107	1.39E-03
GO:0072522	purine-containing compound biosynthetic process	21	21/1131	167/19107	1.40E-03
GO:0031082	BLOC complex	4	4/1131	9/19107	1.41E-03
GO:0031083	BLOC-1 complex	4	4/1131	9/19107	1.41E-03
GO:0044085	cellular component biogenesis	70	70/1131	829/19107	1.90E-03
GO:1901135	carbohydrate derivative metabolic process	53	53/1131	580/19107	2.10E-03
GO:0009058	biosynthetic process	288	288/1131	4154/19107	2.38E-03
GO:0022607	cellular component assembly	62	62/1131	728/19107	2.65E-03
GO:0006164	purine nucleotide biosynthetic process	19	19/1131	154/19107	2.81E-03
GO:0009163	nucleoside biosynthetic process	16	16/1131	123/19107	3.03E-03
GO:0042455	ribonucleoside biosynthetic process	16	16/1131	123/19107	3.03E-03
GO:1901659	glycosyl compound biosynthetic process	16	16/1131	123/19107	3.03E-03
GO:0031099	regeneration	4	4/1131	11/19107	3.05E-03
GO:0042246	tissue regeneration	4	4/1131	11/19107	3.05E-03
GO:0048589	developmental growth	4	4/1131	11/19107	3.05E-03
GO:0070070	proton-transporting V-type ATPase complex assembly	4	4/1131	11/19107	3.09E-03
GO:0070072	vacuolar proton-transporting V-type ATPase complex assembly	4	4/1131	11/19107	3.09E-03
GO:0008440	inositol-1,4,5-trisphosphate 3-kinase activity	4	4/1131	11/19107	3.77E-03
GO:0017134	fibroblast growth factor binding	2	2/1131	2/19107	3.78E-03
GO:0042451	purine nucleoside biosynthetic process	15	15/1131	116/19107	4.13E-03

GO:0046129	purine ribonucleoside biosynthetic process	15	15/1131	116/19107	4.13E-03
GO:0009152	purine ribonucleotide biosynthetic process	18	18/1131	149/19107	4.39E-03
GO:0009260	ribonucleotide biosynthetic process	18	18/1131	149/19107	4.39E-03
GO:0046390	ribose phosphate biosynthetic process	18	18/1131	149/19107	4.39E-03
GO:0004619	phosphoglycerate mutase activity	2	2/1131	2/19107	4.46E-03
GO:0046537	2,3-bisphosphoglycerate-independent phosphoglycerate mutase activity	2	2/1131	2/19107	4.46E-03
GO:0009101	glycoprotein biosynthetic process	18	18/1131	156/19107	4.48E-03
GO:0009100	glycoprotein metabolic process	18	18/1131	157/19107	4.83E-03
GO:0044249	cellular biosynthetic process	272	272/1131	3954/19107	4.90E-03
GO:0055065	metal ion homeostasis	9	9/1131	55/19107	4.92E-03
GO:0000413	protein peptidyl-prolyl isomerization	5	5/1131	21/19107	5.15E-03
GO:0003755	peptidyl-prolyl cis-trans isomerase activity	5	5/1131	21/19107	5.15E-03
GO:0016859	cis-trans isomerase activity	5	5/1131	21/19107	5.15E-03
GO:0018208	peptidyl-proline modification	5	5/1131	21/19107	5.15E-03
GO:0050801	ion homeostasis	9	9/1131	56/19107	5.48E-03
GO:0055080	cation homeostasis	9	9/1131	56/19107	5.48E-03
GO:0040007	growth	12	12/1131	88/19107	5.48E-03
GO:0070071	proton-transporting two-sector ATPase complex assembly	4	4/1131	13/19107	5.51E-03
GO:0009165	nucleotide biosynthetic process	22	22/1131	201/19107	6.36E-03
GO:1901293	nucleoside phosphate biosynthetic process	22	22/1131	201/19107	6.36E-03
GO:1901576	organic substance biosynthetic process	275	275/1131	4024/19107	6.55E-03
GO:0031224	intrinsic to membrane	231	231/1131	3383/19107	6.56E-03
GO:0016286	small conductance calcium-activated potassium channel activity	3	3/1131	7/19107	6.61E-03
GO:0051766	inositol trisphosphate kinase activity	4	4/1131	13/19107	6.73E-03
GO:0006813	potassium ion transport	15	15/1131	125/19107	7.60E-03
GO:0006029	proteoglycan metabolic process	3	3/1131	7/19107	7.62E-03
GO:0015012	heparan sulfate proteoglycan biosynthetic process	3	3/1131	7/19107	7.62E-03

GO:0030166	proteoglycan biosynthetic process	3	3/1131	7/19107	7.62E-03
GO:0030201	heparan sulfate proteoglycan metabolic process	3	3/1131	7/19107	7.62E-03
GO:0072521	purine-containing compound metabolic process	23	23/1131	218/19107	8.09E-03
GO:0071822	protein complex subunit organization	41	41/1131	467/19107	8.65E-03
GO:0008610	lipid biosynthetic process	23	23/1131	218/19107	8.70E-03
GO:0048878	chemical homeostasis	9	9/1131	62/19107	9.20E-03
GO:0005829	cytosol	10	10/1131	74/19107	9.87E-03
GO:0044445	cytosolic part	10	10/1131	74/19107	9.87E-03
GO:0070925	organelle assembly	14	14/1131	119/19107	9.90E-03
GO:0016021	integral to membrane	227	227/1131	3351/19107	1.03E-02
GO:0005956	protein kinase CK2 complex	3	3/1131	8/19107	1.04E-02
GO:0016709	oxidoreductase activity	4	4/1131	14/19107	1.04E-02
GO:0006836	neurotransmitter transport	8	8/1131	50/19107	1.09E-02
GO:0018193	peptidyl-amino acid modification	8	8/1131	52/19107	1.10E-02
GO:0043933	macromolecular complex subunit organization	45	45/1131	533/19107	1.13E-02
GO:0004499	N,N-dimethylaniline monooxygenase activity	3	3/1131	8/19107	1.19E-02
GO:0042555	MCM complex	2	2/1131	3/19107	1.20E-02
GO:0015232	heme transporter activity	2	2/1131	3/19107	1.20E-02
GO:0051260	protein homooligomerization	11	11/1131	85/19107	1.25E-02
GO:0007166	cell surface receptor signaling pathway	83	83/1131	1146/19107	1.26E-02
GO:0009119	ribonucleoside metabolic process	18	18/1131	165/19107	1.27E-02
GO:0006873	cellular ion homeostasis	8	8/1131	53/19107	1.27E-02
GO:0006875	cellular metal ion homeostasis	8	8/1131	53/19107	1.27E-02
GO:0030003	cellular cation homeostasis	8	8/1131	53/19107	1.27E-02
GO:0042278	purine nucleoside metabolic process	17	17/1131	155/19107	1.40E-02
GO:0046128	purine ribonucleoside metabolic process	17	17/1131	155/19107	1.40E-02
GO:0009150	purine ribonucleotide metabolic process	20	20/1131	191/19107	1.46E-02
GO:0009259	ribonucleotide metabolic process	20	20/1131	191/19107	1.46E-02
GO:0019693	ribose phosphate metabolic process	20	20/1131	191/19107	1.46E-02
GO:0006163	purine nucleotide metabolic process	21	21/1131	205/19107	1.49E-02
GO:0000313	organellar ribosome	4	4/1131	18/19107	1.59E-02

GO:0005761	mitochondrial ribosome	4	4/1131	18/19107	1.59E-02
GO:0006461	protein complex assembly	37	37/1131	430/19107	1.59E-02
GO:0070569	uridylyltransferase activity	3	3/1131	9/19107	1.63E-02
GO:0007021	tubulin complex assembly	2	2/1131	4/19107	1.66E-02
GO:0072668	tubulin complex biogenesis	2	2/1131	4/19107	1.66E-02
GO:0050660	flavin adenine dinucleotide binding	11	11/1131	84/19107	1.69E-02
GO:0070271	protein complex biogenesis	37	37/1131	432/19107	1.71E-02
GO:0016843	amine-lyase activity	2	2/1131	4/19107	1.74E-02
GO:0016844	strictosidine synthase activity	2	2/1131	4/19107	1.74E-02
GO:0048037	cofactor binding	35	35/1131	391/19107	1.75E-02
GO:0017004	cytochrome complex assembly	7	7/1131	45/19107	1.75E-02
GO:0019438	aromatic compound biosynthetic process	184	184/1131	2650/19107	1.77E-02
GO:0019887	protein kinase regulator activity	7	7/1131	49/19107	1.80E-02
GO:0016779	nucleotidyltransferase activity	35	35/1131	389/19107	1.80E-02
GO:0055082	cellular chemical homeostasis	8	8/1131	58/19107	1.83E-02
GO:0034645	cellular macromolecule biosynthetic process	221	221/1131	3265/19107	1.84E-02
GO:0090407	organophosphate biosynthetic process	37	37/1131	420/19107	1.85E-02
GO:0065003	macromolecular complex assembly	41	41/1131	493/19107	1.86E-02
GO:0034654	nucleobase-containing compound biosynthetic process	174	174/1131	2499/19107	1.90E-02
GO:0015985	energy coupled proton transport, down electrochemical gradient	10	10/1131	78/19107	1.98E-02
GO:0015986	ATP synthesis coupled proton transport	10	10/1131	78/19107	1.98E-02
GO:0044271	cellular nitrogen compound biosynthetic process	186	186/1131	2692/19107	2.00E-02
GO:0005753	mitochondrial proton-transporting ATP synthase complex	8	8/1131	56/19107	2.02E-02
GO:0015159	polysaccharide transmembrane transporter activity	2	2/1131	4/19107	2.09E-02
GO:0015774	polysaccharide transport	2	2/1131	4/19107	2.09E-02
GO:0033037	polysaccharide localization	2	2/1131	4/19107	2.09E-02
GO:0000917	barrier septum assembly	10	10/1131	80/19107	2.15E-02
GO:0032506	cytokinetic process	10	10/1131	80/19107	2.15E-02
GO:0090529	cell septum assembly	10	10/1131	80/19107	2.15E-02
GO:0043161	proteasomal ubiquitin-dependent protein	4	4/1131	19/19107	2.18E-02

	catabolic process				
GO:0018130	heterocycle biosynthetic process	191	191/1131	2776/19107	2.19E-02
GO:0006457	protein folding	14	14/1131	135/19107	2.20E-02
GO:0000278	mitotic cell cycle	10	10/1131	80/19107	2.23E-02
GO:0000276	mitochondrial proton-transporting ATP synthase complex, coupling factor F(o)	7	7/1131	47/19107	2.28E-02
GO:0008013	beta-catenin binding	4	4/1131	18/19107	2.33E-02
GO:0050797	thymidylate synthase (FAD) activity	2	2/1131	4/19107	2.45E-02
GO:0010498	proteasomal protein catabolic process	4	4/1131	20/19107	2.57E-02
GO:0008150	biological_process	857	857/1131	13890/19107	2.60E-02
GO:0009059	macromolecule biosynthetic process	221	221/1131	3294/19107	2.61E-02
GO:0009142	nucleoside triphosphate biosynthetic process	11	11/1131	95/19107	2.69E-02
GO:0009145	purine nucleoside triphosphate biosynthetic process	11	11/1131	95/19107	2.69E-02
GO:0009201	ribonucleoside triphosphate biosynthetic process	11	11/1131	95/19107	2.69E-02
GO:0009206	purine ribonucleoside triphosphate biosynthetic process	11	11/1131	95/19107	2.69E-02
GO:0006486	protein glycosylation	15	15/1131	149/19107	2.71E-02
GO:0043413	macromolecule glycosylation	15	15/1131	149/19107	2.71E-02
GO:0070085	glycosylation	15	15/1131	149/19107	2.71E-02
GO:0006754	ATP biosynthetic process	10	10/1131	82/19107	2.72E-02
GO:0042132	fructose 1,6-bisphosphate 1-phosphatase activity	2	2/1131	5/19107	3.10E-02
GO:0004888	transmembrane signaling receptor activity	65	65/1131	910/19107	3.17E-02
GO:0004748	ribonucleoside-diphosphate reductase activity, thioredoxin disulfide as acceptor	2	2/1131	5/19107	3.24E-02
GO:0016728	oxidoreductase activity, acting on CH or CH2 groups, disulfide as acceptor	2	2/1131	5/19107	3.24E-02
GO:0051259	protein oligomerization	11	11/1131	98/19107	3.29E-02
GO:0031418	L-ascorbic acid binding	2	2/1131	5/19107	3.32E-02
GO:0043623	cellular protein complex assembly	26	26/1131	299/19107	3.33E-02
GO:0016020	membrane	409	409/1131	6379/19107	3.34E-02
GO:1901362	organic cyclic compound	191	191/1131	2812/19107	3.37E-02

	biosynthetic process				
GO:0045259	proton-transporting ATP synthase complex	9	9/1131	73/19107	3.37E-02
GO:0034062	RNA polymerase activity	21	21/1131	220/19107	3.41E-02
GO:0005315	inorganic phosphate transmem -brane transporter activity	2	2/1131	5/19107	3.42E-02
GO:1901677	phosphate transmembrane transporter activity	2	2/1131	5/19107	3.42E-02
GO:0055086	nucleobase-containing small molecule metabolic process	29	29/1131	331/19107	3.42E-02
GO:0045263	proton-transporting ATP synthase complex, coupling factor F(o)	8	8/1131	62/19107	3.43E-02
GO:0044710	single-organism metabolic process	165	165/1131	2357/19107	3.47E-02
GO:0050662	coenzyme binding	27	27/1131	305/19107	3.52E-02
GO:0042255	ribosome assembly	2	2/1131	5/19107	3.58E-02
GO:0042256	mature ribosome assembly	2	2/1131	5/19107	3.58E-02
GO:0003899	DNA-directed RNA polymerase activity	15	15/1131	146/19107	3.69E-02
GO:0006448	regulation of translational elongation	2	2/1131	5/19107	3.70E-02
GO:0016255	attachment of GPI anchor to protein	2	2/1131	5/19107	3.77E-02
GO:0033043	regulation of organelle organization	10	10/1131	88/19107	3.78E-02
GO:0000280	nuclear division	8	8/1131	65/19107	3.81E-02
GO:0007067	mitosis	8	8/1131	65/19107	3.81E-02
GO:0034622	cellular macromolecular complex assembly	30	30/1131	362/19107	3.95E-02
GO:0016791	phosphatase activity	21	21/1131	224/19107	3.95E-02
GO:0005143	interleukin-12 receptor binding	1	1/1131	1/19107	3.96E-02
GO:0030031	cell projection assembly	6	6/1131	43/19107	3.96E-02
GO:0006480	N-terminal protein amino acid methylation	2	2/1131	6/19107	4.02E-02
GO:0031365	N-terminal protein amino acid modification	2	2/1131	6/19107	4.02E-02
GO:0005506	iron ion binding	17	17/1131	179/19107	4.04E-02
GO:0009116	nucleoside metabolic process	19	19/1131	203/19107	4.07E-02
GO:1901657	glycosyl compound metabolic process	19	19/1131	203/19107	4.07E-02
GO:0044425	membrane part	263	263/1131	4039/19107	4.10E-02
GO:0043412	macromolecule modification	128	128/1131	1800/19107	4.14E-02
GO:0015936	coenzyme A metabolic process	3	3/1131	13/19107	4.17E-02

GO:0034470	ncRNA processing	20	20/1131	217/19107	4.19E-02
GO:0046034	ATP metabolic process	10	10/1131	88/19107	4.20E-02
GO:0043022	ribosome binding	3	3/1131	13/19107	4.26E-02
GO:0006721	terpenoid metabolic process	5	5/1131	31/19107	4.45E-02
GO:0016114	terpenoid biosynthetic process	5	5/1131	31/19107	4.45E-02
GO:0004872	receptor activity	73	73/1131	1048/19107	4.52E-02
GO:0044281	small molecule metabolic process	69	69/1131	917/19107	4.59E-02
GO:0019203	carbohydrate phosphatase activity	2	2/1131	6/19107	4.62E-02
GO:0050308	sugar-phosphatase activity	2	2/1131	6/19107	4.62E-02
GO:0005890	sodium:potassium-exchanging ATPase complex	3	3/1131	15/19107	4.90E-02
GO:0051128	regulation of cellular component organization	14	14/1131	143/19107	4.90E-02
GO:0044260	cellular macromolecule metabolic process	379	379/1131	5878/19107	4.96E-02
