# Supplementary figures and images for: Climate Adaptation and Drift Shape the Genomes of Two Eel-Goby Sister Species Endemic to Contrasting Latitude
Source: Animals (Basel). 2023 Oct 17;13(20):3240. doi: 10.3390/ani13203240 (PMC10603712; doi:10.3390/ani13203240)

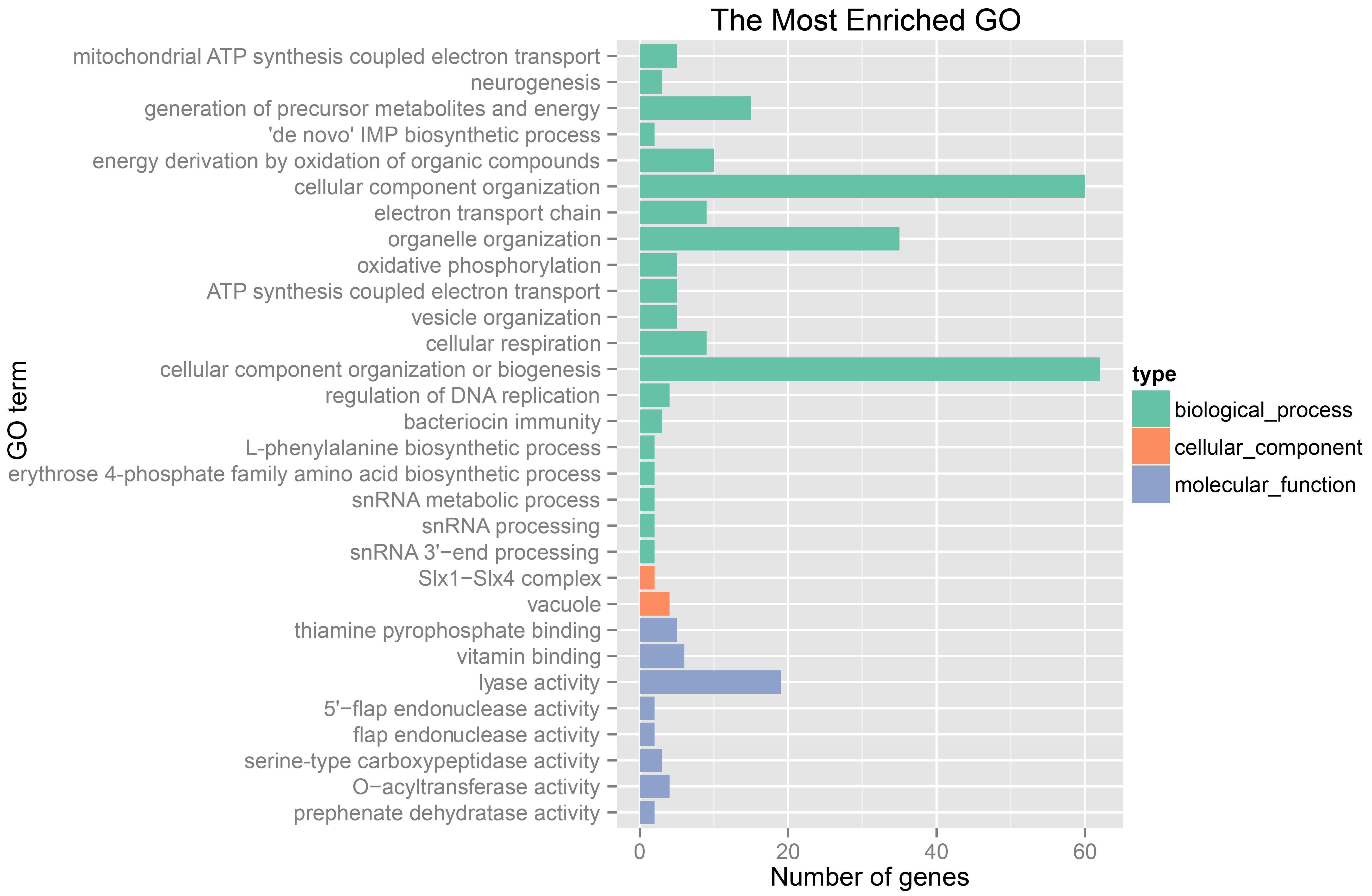

Supplement: Supplementary file 1 [file animals-13-03240-s001.zip › Supplementary figures/Figure.S2.jpg]

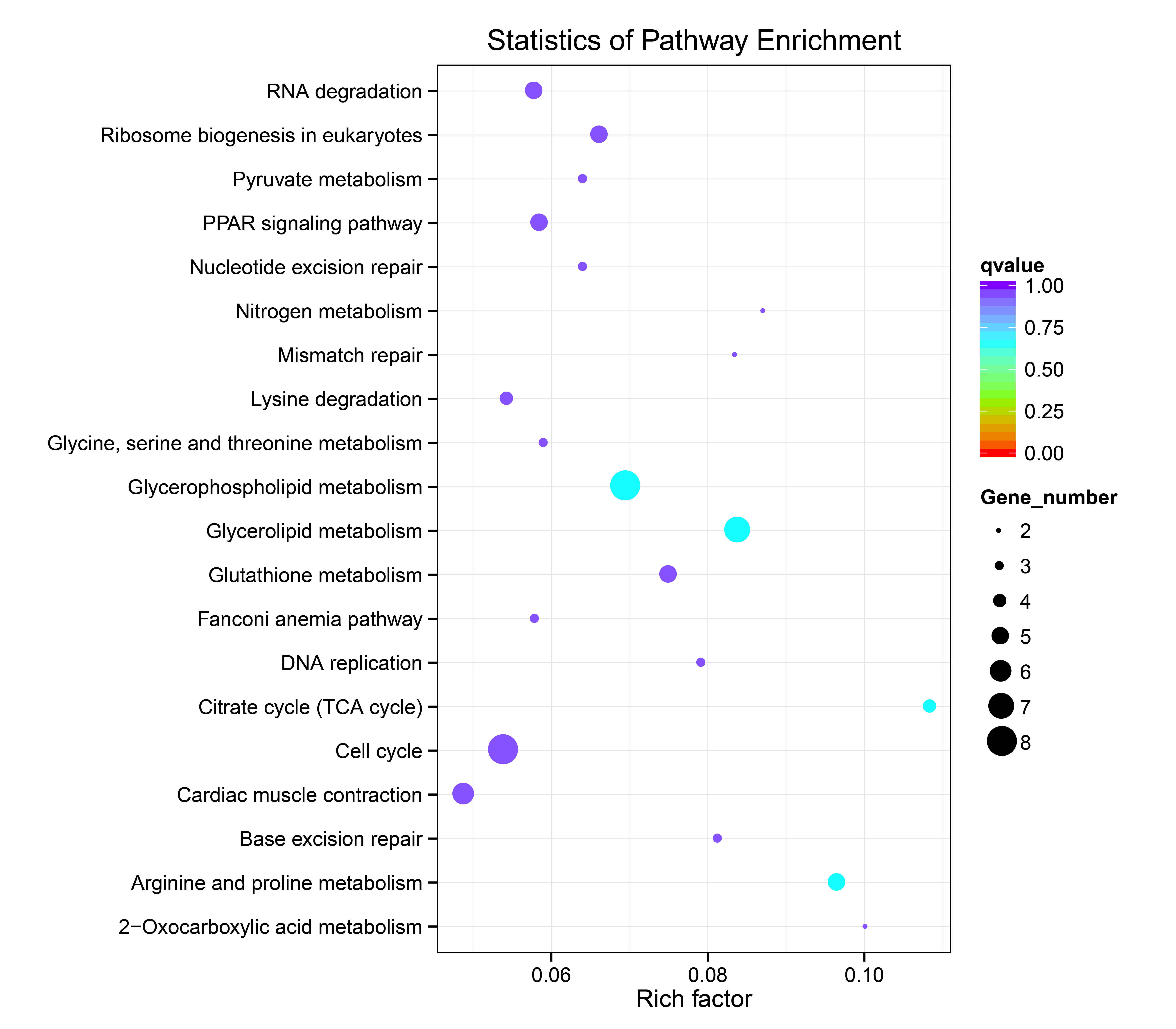

Supplement: Supplementary file 1 [file animals-13-03240-s001.zip › Supplementary figures/Figure.S3.jpg]

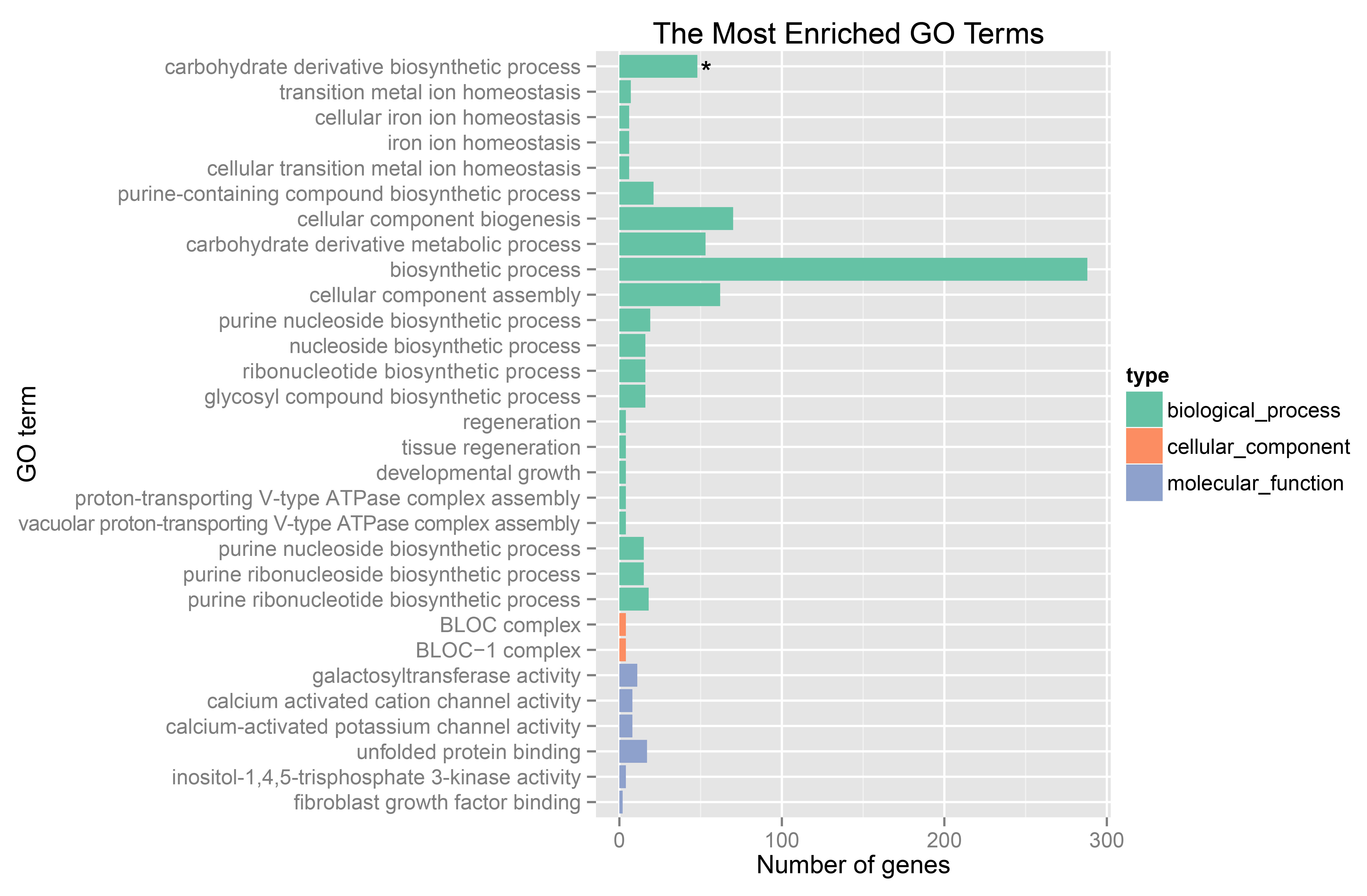

Supplement: Supplementary file 1 [file animals-13-03240-s001.zip › Supplementary figures/Figure.S4.jpg]

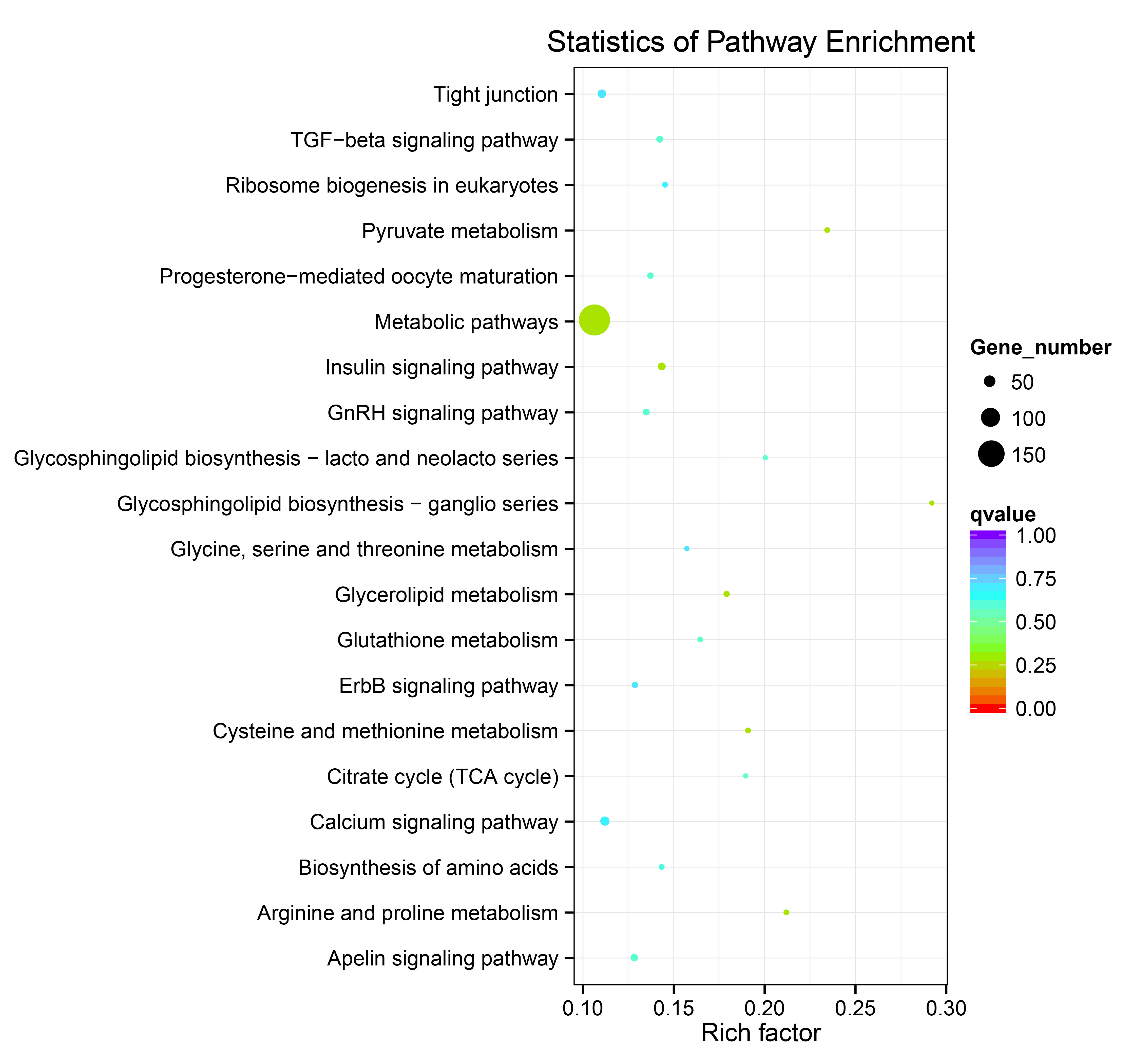

Supplement: Supplementary file 1 [file animals-13-03240-s001.zip › Supplementary figures/Figure.S5.jpg]
